# UBN1/2 of HIRA complex is responsible for recognition and deposition of H3.3 at *cis*-regulatory elements of genes in mouse ES cells

**DOI:** 10.1186/s12915-018-0573-9

**Published:** 2018-10-03

**Authors:** Chaoyang Xiong, Zengqi Wen, Juan Yu, Jun Chen, Chao-Pei Liu, Xiaodong Zhang, Ping Chen, Rui-Ming Xu, Guohong Li

**Affiliations:** 1 National Laboratory of Biomacromolecules, CAS Center for Excellence in Biomacromolecules, Institute of Biophysics, Chinese Academy of Sciences, Beijing, 100101 China; 2 University of Chinese Academy of Sciences, Beijing, 100049 China; 3 College of Life Sciences, Wuhan University, Wuhan, 430072 China

**Keywords:** Histone variant H3.3, HIRA complex, UBN1, UBN2, Enhancer

## Abstract

**Background:**

H3.3 is an ancient and conserved H3 variant and plays essential roles in transcriptional regulation. HIRA complex, which is composed of HIRA, UBN1 or UBN2, and Cabin1, is a H3.3 specific chaperone complex. However, it still remains largely uncharacterized how HIRA complex specifically recognizes and deposits H3.3 to the chromatin, such as promoters and enhancers.

**Results:**

In this study, we demonstrate that the UBN1 or UBN2 subunit is mainly responsible for specific recognition and direct binding of H3.3 by the HIRA complex. While the HIRA subunit can enhance the binding affinity of UBN1 toward H3.3, Cabin1 subunit cannot. We also demonstrate that both Ala87 and Gly90 residues of H3.3 are required and sufficient for the specific recognition and binding by UBN1. ChIP-seq studies reveal that two independent HIRA complexes (UBN1-HIRA and UBN2-HIRA) can cooperatively deposit H3.3 to *cis*-regulatory regions, including active promoters and active enhancers in mouse embryonic stem (mES) cells. Importantly, disruption of histone chaperone activities of UBN1 and UBN2 by FID/AAA mutation results in the defect of H3.3 deposition at promoters of developmental genes involved in neural differentiation, and subsequently causes the failure of activation of these genes during neural differentiation of mES cells.

**Conclusion:**

Together, our results provide novel insights into the mechanism by which the HIRA complex specifically recognizes and deposits H3.3 at promoters and enhancers of developmental genes, which plays a critical role in neural differentiation of mES cells*.*

**Electronic supplementary material:**

The online version of this article (10.1186/s12915-018-0573-9) contains supplementary material, which is available to authorized users.

## Background

In eukaryotic cells, the genomic DNA is hierarchically compacted into chromatin to fit inside the nucleus. The 3D organization and dynamics of chromatin fibers play a central role in regulation of gene transcription and other biological processes involving DNA, such as DNA replication, repair, and recombination [1]. The nucleosome is the basic repeating structural unit of chromatin [2], which is composed of an octamer of histones with two copies of each H2A, H2B, H3 and H4, and 147 base pairs of DNA wrapped around the histone octamer with about 1.7 superhelical turns in a left-handed manner [3]. These histone proteins are called canonical histones, which are synthesized and assembled into the chromatin in a DNA replication-dependent manner at S phase during the cell cycle. To date, more than a dozen of variants of the canonical histones have been identified for H2A, H2B and H3, but not for H4. The differences between histone variants and their canonical counterparts range from several amino acid residues to an entire new structural domain. Histone variants are synthesized and incorporated into chromatin throughout the cell cycle [4]. Histone chaperones selectively bind histones, either to ensure their stability or to assemble into chromatin [5]. Histone chaperones are essential in the regulation of histone dynamics, genome stability and cell identity [6]. The incorporation of histone variants may create architecturally distinct chromatin regions by regulating the structure and dynamics of chromatin fibers, which enables the chromatin to play diverse functions in chromatin-associated processes [7, 8].

The canonical histone H3 is exclusively recognized and assembled to newly synthesized DNA by CAF-1 (chromatin assembly factor 1) complex, which is a histone chaperone complex specific to H3 [9]. H3.3, an evolutionary conserved histone variant of the H3, is constitutively expressed throughout the whole cell cycle [10] and is deposited to chromatin in a DNA synthesis-independent manner [11]. Two histone chaperone complexes, namely HIRA (histone regulator A) complex [9] and DAXX/ATRX (death domain-associated protein/α-thalassemia/mental retardation syndrome X-linked) complex [12, 13], have been shown to specifically recognize and deposit H3.3 to the chromatin. HIRA complex deposits H3.3 mainly at euchromatin regions such as promoters and actively transcribed gene bodies [12], whereas DAXX/ATRX deposits H3.3 at pericentric and telomeric heterochromatin regions [12–14].

The H3.3 variant differs from H3.1 by only five amino acid residues: Ala87, Ile89, Gly90, Ser96, which are all hidden inside the nucleosome core particle, and Ser31, which is exposed outside of the nucleosome core particle [15]. However, a single replacement of “S” with “A” at position 31 of the H3.3 have no effect on the deposition pathway, suggesting that H3.3 Ser31 and its phosphorylation do not play a role in H3.3 deposition [16]. Most recently, we and others showed that Ala87 and Gly90 are the principal determinants of H3.3 specificity in DAXX recognition [17, 18]. DAXX uses a shallow hydrophobic pocket to accommodate the small hydrophobic Ala87 of H3.3, and a polar-binding environment of DAXX prefers Gly90 of H3.3 to the hydrophobic Met90 of H3.1 [17, 18].

The HIRA complex is an evolutionarily conserved chaperone. In mammalian cells, it is composed of three core subunits: HIRA, UBN1 (Ubinuclein 1), and Cabin1 (calcineurin-binding protein 1) [9, 19]. The HIRA subunit is orthologous to yeast Hir1p and Hir2p [20]. UBN1 is orthologous to Hpc2p of yeast, with a paralog—UBN2 in mammals [19, 21], and Cabin1 is orthologous to Hir3p of yeast [22]. HIRA subunit associates with HRD domain of UBN1 through its WD repeat domain [19] and interacts with the N terminus of Cabin1 through its C terminus [22]. Thus, HIRA protein acts as a scaffold to bring together UBN1 and Cabin1. HIRA complex and H3.3 take parts in various stages of embryo development, including gametogenesis, fertilization, early embryonic development, and tissue formation [23–25], which suggests that the proper distribution of H3.3 at the chromatin landscape is important during development. Recently, mutations in H3.3 have been reported in cancers, such as K27M mutation in pediatric high-grade glioma [26–28] and K36M mutation in skeletal neoplasm [29]. Further studies revealed that these mutations reprogram the epigenetic landscape of tumor cells in a gain-of-function manner to promote tumorigenesis [30–34]. Thus, how HIRA complex specifically recognizes H3.3 and regulates the deposition of H3.3 across the genome is fundamental for the mechanistic understanding of the function of HIRA complex and H3.3 under both physiological and pathological conditions.

It has recently been reported that UBN1 Hpc2-related domain (HRD) binds H3.3 specifically independent of HIRA subunit in vitro [35], it remains unclear what are the functions of other subunits of HIRA complex in H3.3 chaperoning. In addition, we previously found that H3.3 is also highly enriched at enhancers, to prime for the activation of RAR/RXR targeted genes [36]. However, little is known how the deposition of H3.3 at enhancers is regulated. Here, we demonstrated that in addition to UBN1, UBN2 also binds H3.3 specifically and directly, and HIRA subunit can enhance the binding between UBN1 and H3.3. Genome-wide ChIP-seq analyses further showed that two independent HIRA complexes (UBN1-HIRA and UBN2-HIRA) were found to cooperatively deposit H3.3 at *cis-*regulatory regions, including active promoters and active enhancers, in mouse embryonic stem (mES) cells. Impairment of H3.3 deposition at promoters of developmental genes by FID/AAA mutations of UBN1 and UBN2 disrupts chaperone activity of UBN1 and UBN2 in mES cells and causes the failure of activation of these genes during neural differentiation of mES cells.

## Results

### Both UBN1 and UBN2 subunits are responsible for the specific and direct binding of H3.3 by HIRA complex

**Fig. 1:**
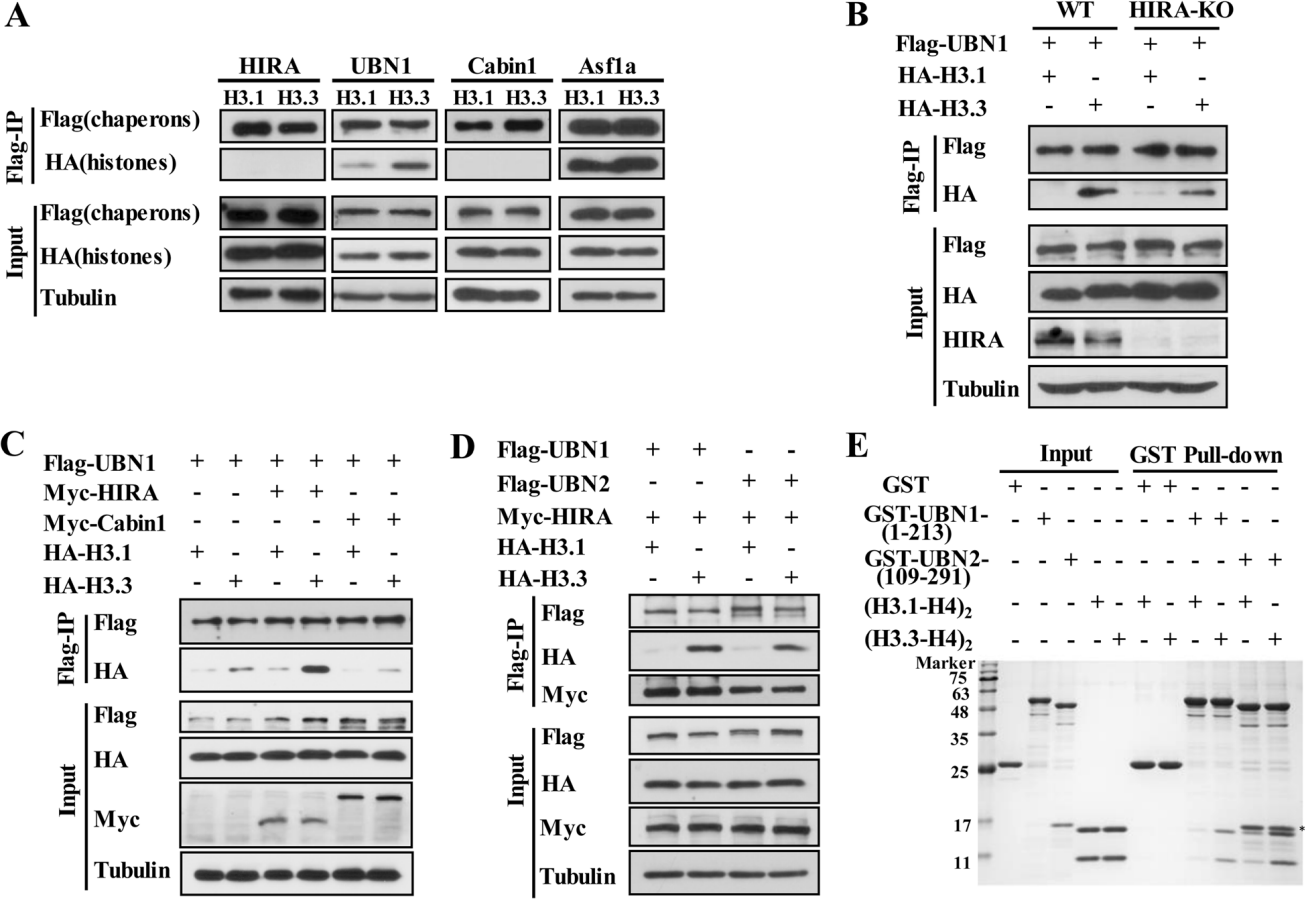
UBN1 and UBN2 are responsible for the specific recognition and direct binding of H3.3. **a** UBN1 specifically recognizes H3.3. Interaction between HIRA, UBN1, Cabin1, Asf1a, and H3.1, H3.3 is analyzed by Western blot analysis of anti-Flag immunoprecipitates. **b** HIRA is dispensable for the interaction between UBN1 and H3.3. The interaction between UBN1 and H3.3 is analyzed by immunoprecipitation in wild type (WT) or HIRA knockout (HIRA KO) HEK293T cells as illustrated. **c** HIRA enhances the interaction between UBN1 and H3.3. The effect of HIRA and Cabin1 on the interaction between UBN1 and H3.3 is analyzed by Western blot analysis of anti-Flag immunoprecipitates. **d** Both UBN1 and UBN2 specifically interact with H3.3. Interaction between UBN1, UBN2, and H3.1, H3.3 in the presence of HIRA is analyzed by Western blot analysis of anti-Flag immunoprecipitates. **e** The direct interaction between UBN1 (aa1-213), UBN2 (aa109-291), and (H3.1-H4)_2_, (H3.3-H4)_2_ tetramers is analyzed by GST pull-down followed by Coomassie staining. Input corresponds to 10% proteins. * indicates nonspecific band in GST-UBN2-(aa109-291) sample

It was reported that UBN1 interacted with H3.3 directly in vitro [35]. However, knockdown of HIRA subunit also impaired the deposition of H3.3 [37, 38]. To further investigate the molecular mechanism by which the HIRA complex recognizes and binds H3.3 in detail, we detected the interaction between the subunits of HIRA complex and H3.3 by co-immunoprecipitation (co-IP). As shown in Fig. 1a, Flag-UBN1, but not Flag-HIRA or Flag-Cabin1, specifically interacts with HA-H3.3 in HEK293T cells. Interestingly, Flag-UBN1 still preferentially interacts with HA-H3.3 after HIRA knockout in HEK293T cells (Fig. 1b). These results suggested that the interaction between UBN1 and H3.3 was independent of HIRA. Moreover, we found that the HIRA subunit, but not Cabin1, enhances the specificity and affinity of UBN1 to H3.3 (Fig. 1c).

Apart from UBN1, there is another yeast Hpc2p homology in mammals called UBN2 [19, 21]. Similar to UBN1, UBN2 also contains the conserved HRD domain at its N-terminus, which is important for the interaction with H3.3 (Additional file 1: Figure S1). We found that Flag-UBN2 also binds to HA-H3.3 specifically as Flag-UBN1 does (Fig. 1d). GST pull-down also confirmed that both GST-UBN1 and GST-UBN2 fusion proteins that contain the HRD domain preferentially bind to (H3.3-H4)_2_ tetramers compared with (H3.1-H4)_2_ tetramers (Fig. 1e). Together, these results suggested that both UBN1- and UBN2-HIRA complexes may mediate the deposition of H3.3 in mammal cells.

### UBN1 mediates the interaction between HIRA subunit and histone variant H3.3

**Fig. 2:**
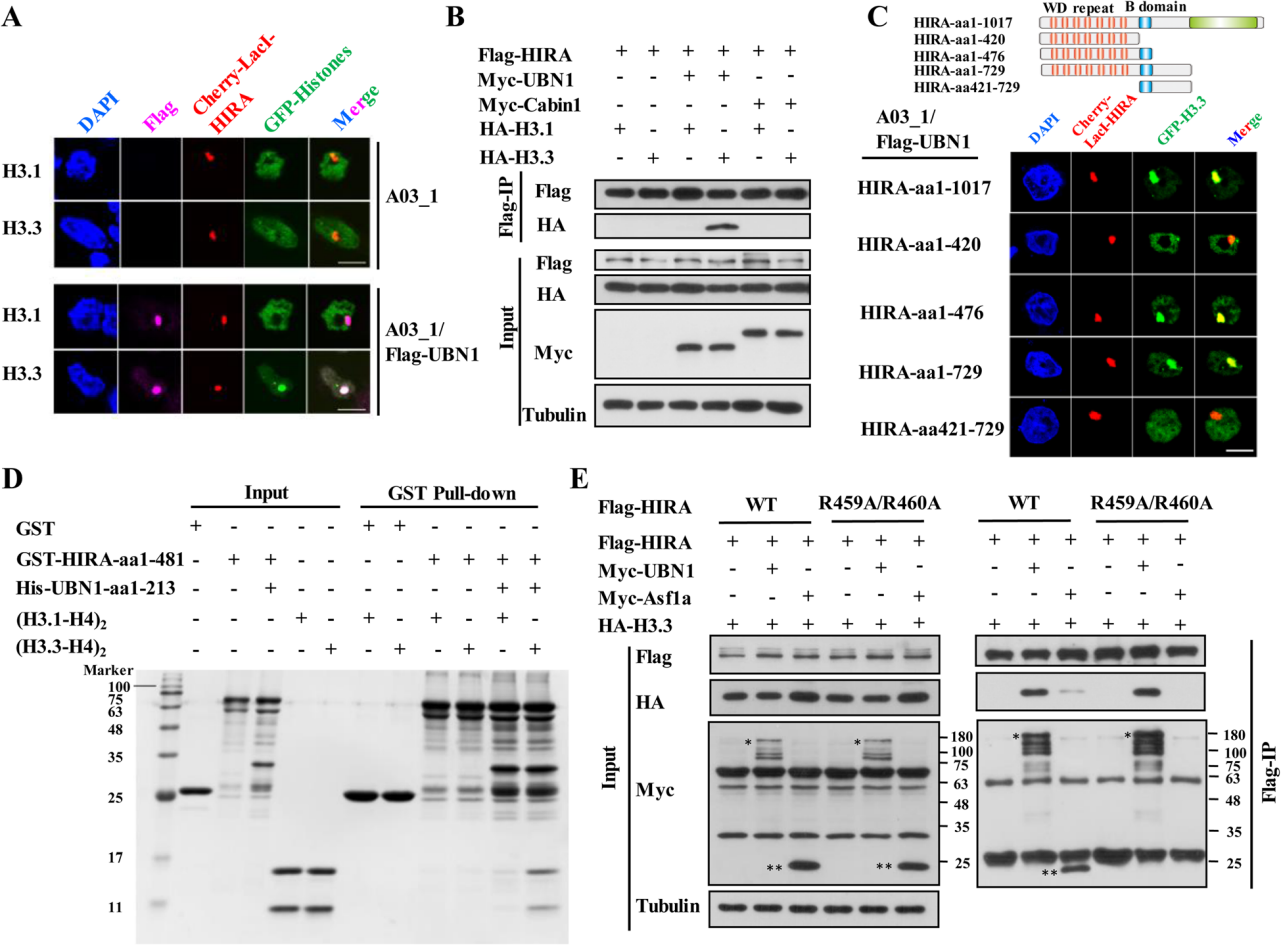
UBN1 mediates the interaction between HIRA subunit and histone variant H3.3. **a** UBN1 mediates the specific interaction between HIRA and H3.3. Interaction between HIRA subunit and H3.3 is analyzed by LacO-LacI targeting system in A03_1 or A03_1/Flag-UBN1 cell lines. A green focus indicates the interaction between the histones with chaperones. Scale bar, 10 μm. **b** Interaction between HIRA subunit and H3.3 is analyzed in the presence of UBN1 or Cabin1 by Western blot analysis of anti-Flag immunoprecipitates. **c** Both WD repeat domain and B domain of HIRA are involved in the interaction with H3.3. Top panel, schematic presentation of full length and truncation mutants of human HIRA; bottom panel, interaction between truncation mutants of HIRA subunit and H3.3 is analyzed in A03_1/Flag-UBN1 cell line. Statistic results are shown in Additional file 1: Figure S2D. Scale bar, 10 μm. **d** The effect of UBN1 (aa1-213) on the interaction between HIRA (aa1-481) and H3.3 is analyzed by GST pull-down followed by Coomassie staining. Input corresponds to 10% proteins. **e** The interaction between HIRA subunit and H3.3 is independent of Asf1a. The effect of Asf1a on the interaction between HIRA subunit and H3.3 is analyzed by Western blot analysis of anti-Flag immunoprecipitates. * indicates Myc-UBN1; ** indicates Myc-Asf1a

Although the HIRA subunit did not interact with H3.3 directly, previous studies indicated that the HIRA subunit contributes to H3.3 deposition [12, 38]. To understand the exact function of HIRA subunit in H3.3 recognition and binding, we performed LacO-LacI targeting assay in wild type (WT) A03_1 cell line or A03_1/Flag-UBN1 (which stably expresses Flag-UBN1) cell line (Additional file 1: Figure S2A and S2B) [39]. As illustrated in Additional file 1: Figure S2A, in the LacO-LacI targeting system, a LacO repeat is integrated in the genome of A03_1 hamster cell line, and then the LacI-cherry-tagged protein and the GFP-tagged protein are co-expressed in the cells. The LacI-cherry-tagged protein is recruited to the LacO locus by LacI tag, resulting in a red spot. If the GFP-tagged protein can interact with the LacI-cherry-tagged protein, it will also be recruited to the LacO locus, resulting in a green spot. Interestingly, we found that Cherry-LacI-HIRA fusion protein strongly co-localized with GFP-H3.3 in the A03_1/Flag-UBN1 cells, but not in WT A03_1 cells (Fig. 2a), suggesting that UBN1 indeed mediated the interaction between HIRA subunit and H3.3. Our co-IP experiments further confirmed that Flag-HIRA subunit cannot bind to HA-H3.3 in the absence of Myc-UBN1 and showed that Myc-Cabin1 fails to mediate the interaction between Flag-HIRA and HA-H3.3 (Fig. 2b). To analyze why endogenous UBN1 cannot complement the interaction between exogenous HIRA and H3.3, we compared the exogenous and endogenous protein levels of HIRA and UBN1 in HEK293T cells. As shown in Additional file 1: Figure S2C, the exogenously expressed HIRA is much more abundant than endogenous HIRA, thus endogenous UBN1 is not sufficient to mediate the interaction between exogenous HIRA and H3.3. When UBN1 is overexpressed, we also see that exogenous UBN1 is much more abundant than endogenous UBN1, thus exogenous UBN1 can effectively mediate the interaction between exogenous HIRA and H3.3 (Additional file 1: Figure S2C). Taken together, these results demonstrated that UBN1 mediates the interaction between the HIRA subunit and histone variant H3.3.

To identify the regions of the HIRA subunit involved in interactions with UBN1 and H3.3, we generated a series of truncated proteins containing either the WD repeat domain (aa1–352) or B domain (aa421–479) of HIRA (Fig. 2c), and performed the LacO-LacI targeting assay in the A03_1/Flag-UBN1 cells. Although the WD repeat domain of HIRA protein is sufficient to bind UBN1 [19], it is not sufficient for the interaction with H3.3 (Fig. 2c, Additional file 1: Figure S2D). Instead, we found that both the WD repeat domain and the B domain are required for the interaction between Cherry-LacI-HIRA and GFP-H3.3 (Fig. 2c, Additional file 1: Figure S2D). Co-IP analyses of Flag-tagged HIRA truncations and HA-H3.3 in HEK293T cells further confirmed this result (Additional file 1: Figure S2E). In addition, in vitro GST pull-down experiments demonstrated that GST-HIRA-aa1-481 (containing the WD repeat domain and B domain) cannot bind (H3.1-H4)_2_ or (H3.3-H4)_2_ tetramers, whereas the UBN1 (His-UBN1-aa1–213) evidently and specifically promotes the interaction between GST-HIRA-aa1-481 and (H3.3-H4)_2_ tetramers (Fig. 2d), further supporting that UBN1 mediates the interaction between the HIRA subunit and H3.3. It has been reported that the B domain of HIRA is involved in binding Asf1a, and the Arg459 and Arg460 residues of HIRA are required for this interaction [40]. As Asf1a also can bind to H3.3 [9], thus it is possible that Asf1a also takes part in the interaction between HIRA and H3.3. To this end, we performed co-IP analyses for the interaction between HIRA and H3.3 using a HIRA-R459A/R460A mutant, which abolishes the interaction between HIRA and Asf1a. We found that the Flag-HIRA-R459A/R460A mutant still effectively interacted with HA-H3.3 in the presence of UBN1 (Fig. 2e). Thus, the B domain of HIRA contributes to UBN1-mediated interaction between HIRA and H3.3, which is not dependent on Asf1a.

### Residues Ala87 and Gly90 of H3.3 are important for recognition and binding of H3.3 by HIRA complex

**Fig. 3:**
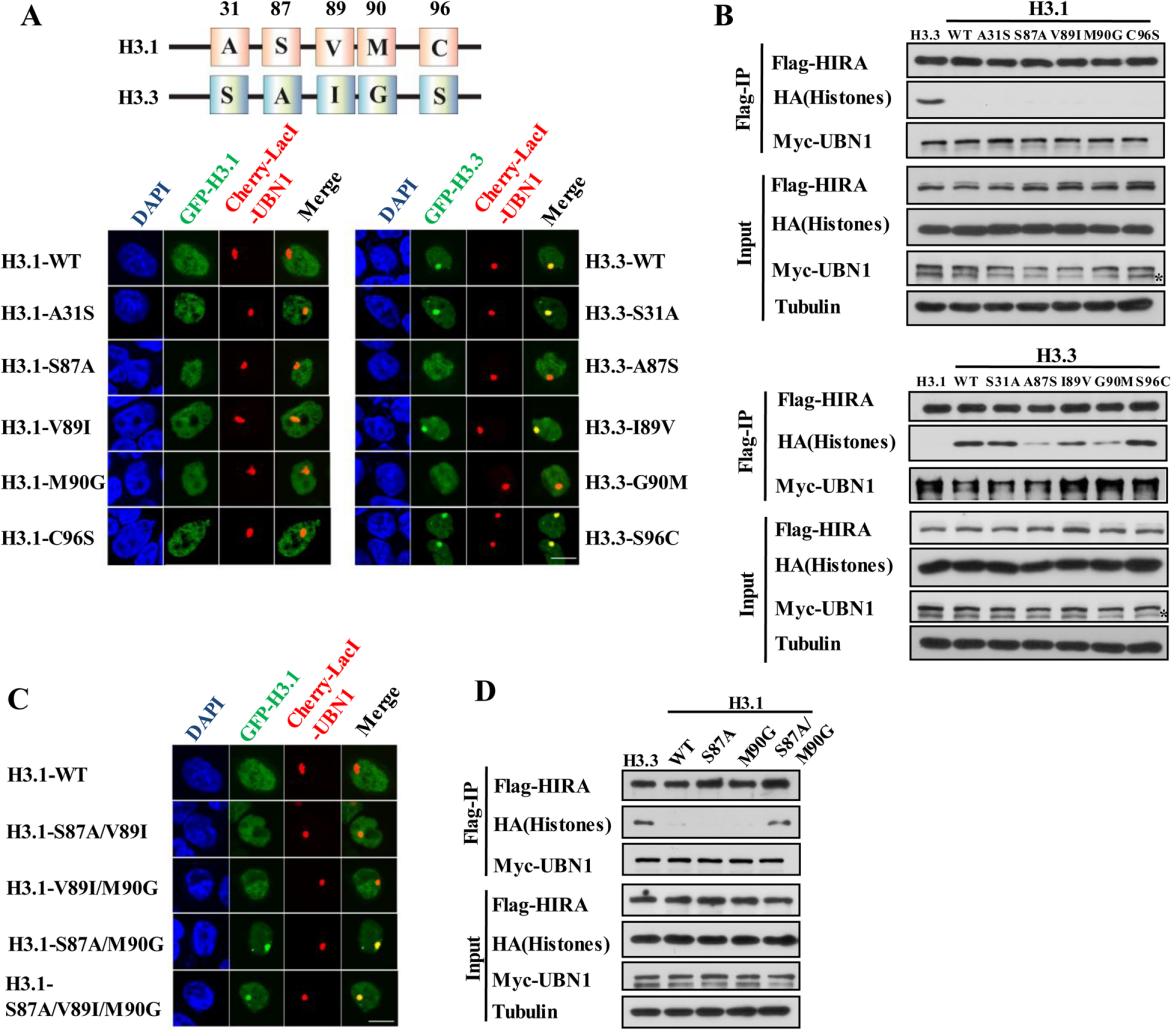
Residues Ala87 and Gly90 of H3.3 are important for recognition and binding of H3.3 by HIRA complex. **a**, **b** Both Ala87 and Gly90 of H3.3 are required for binding UBN1. Top panel, schematic diagram shows the different amino acid residues between H3.1 and H3.3; Bottom panel, interaction between UBN1 subunit and H3.1 or H3.3 mutants is analyzed by LacO-LacI targeting system (**a**) or Western blot of anti-Flag immunoprecipitates (**b**). Statistic results are shown in Additional file 1: Figure S3C. Scale bar, 10 μm. (**c**, **d**) Ala87 and Gly90 of H3.3 are sufficient to confer the specificity toward UBN1. Interaction between UBN1 subunit and H3.1 mutants is analyzed by LacO-LacI targeting system (**c**) and Western blot of anti-Flag immunoprecipitates (**d**). Statistic results are shown in Additional file 1: Figure S3D, Scale bar, 10 μm

Histone variant H3.3 differs from canonical H3.1 by only five amino acids: Ser31, Ala87, Ile89, Gly90, and Ser96 (Fig. 3a). Previously, we have shown that either Ala87 or Gly90 residue of H3.3 is sufficient for the recognition of H3.3 by DAXX (Additional file 1: Figure S3A,S3B) [18]. We therefore wondered which of the five residue(s) is(are) involved in the recognition and binding of H3.3 by UBN1. Thus, we explored the key residues by LacO-LacI targeting assays and co-IP experiments in vivo*.* We found that either A87S or G90M mutation of H3.3 abrogated its interaction with UBN1 (Fig. 3a, b, Additional file 1: Figure S3C). These data suggested that both Ala87 and Gly90 residues of H3.3 are necessary for the recognition and binding of UBN1. However, neither H3.1-S87A nor H3.1-M90G mutant gained the ability to bind UBN1 (Fig. 3a, b, Additional file 1: Figure S3C). We further made H3.1 double and triple mutations toward H3.3 and then monitored the interaction between these H3.1 mutants and UBN1. As shown in Fig. 3c and Fig. 3d, UBN1 now can efficiently interact with H3.1 mutants containing both S87A and M90G mutations. Quantitative analyses showed that S87A/M90G double mutations significantly enhanced the binding affinity of H3.1 for UBN1 (Additional file 1: Figure S3D). Taken together, these results revealed that both Ala87 and Gly90 residues of H3.3 are required and sufficient for the recognition and binding by the HIRA complex.

### UBN1 and UBN2 cooperatively deposit H3.3 at *cis*-regulatory elements in mES cells

**Fig. 4:**
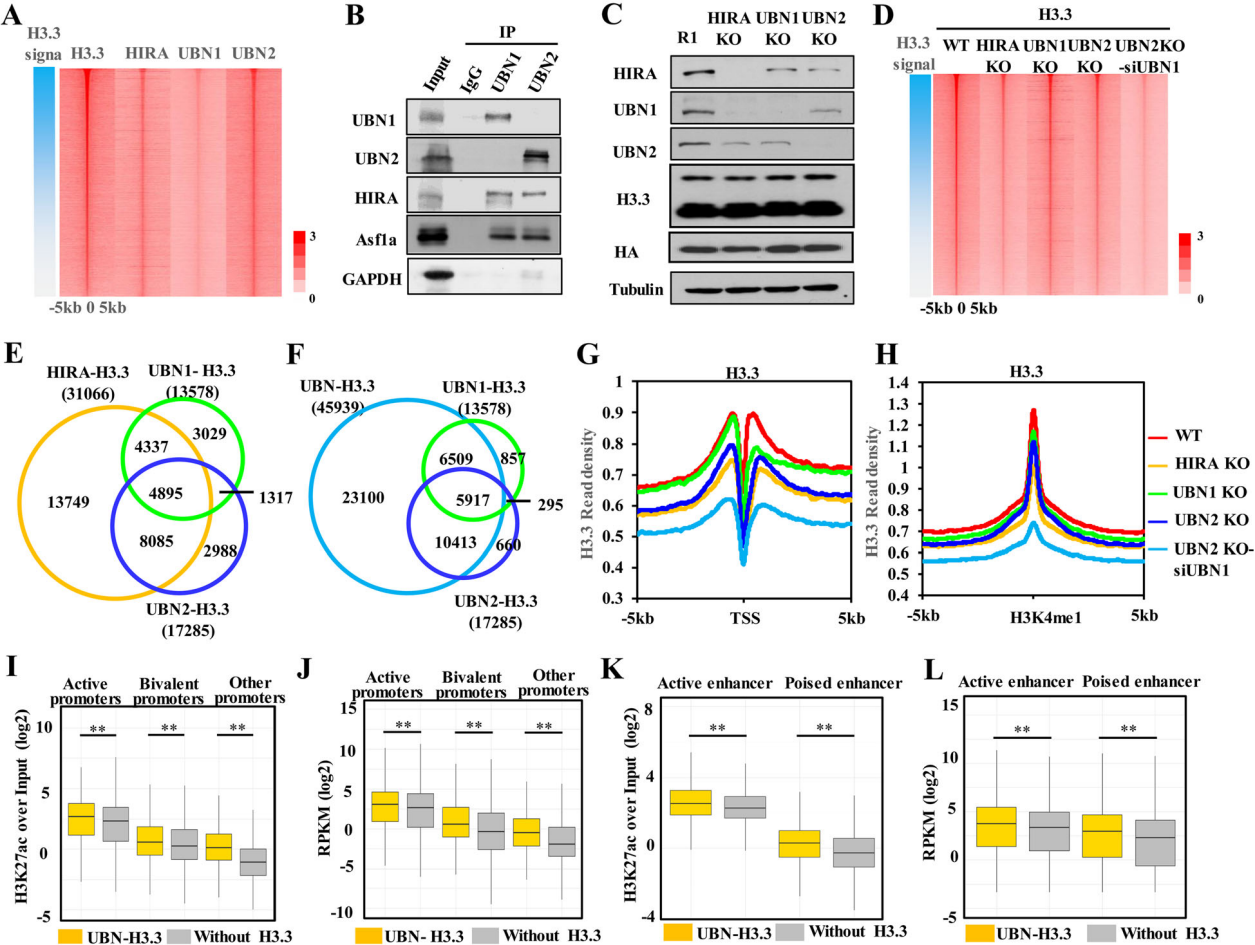
UBN1 and UBN2 cooperatively deposit H3.3 at *cis-*regulatory elements in mESC. **a** Heat map shows that H3.3, HIRA, UBN1, and UBN2 are well co-localized at H3.3 peaks. H3.3 peaks overlapping with UBN1 or UBN2 are sorted descendingly according to the reads density of H3.3. Read density of H3.3, UBN1, UBN2, and HIRA are counted around 5 kb of H3.3 peak center. **b** UBN1 and UBN2 do not interact with each other. The endogenous interaction between UBN1 and UBN2 is analyzed by Western blot analysis of anti-UBN1 and anti-UBN2 immunoprecipitates. **c** Western blot shows the proteins levels of HIRA, UBN1, UBN2, and H3.3 in HIRA KO, UBN1 KO, and UBN2 KO cell lines. H3.3 antibody recognizes both 3XFlag-HA knock-in H3.3 and none tagged H3.3. HA antibody recognizes 3XFlag-HA knock-in H3.3. **d** Heat map shows H3.3 reads density in WT, HIRA KO, UBN1 KO, UBN2 KO, and UBN2 KO-siUBN1 cells. **e** Venn diagram shows the overlapping among HIRA-H3.3, UBN1-H3.3, and UBN2-H3.3. **f** Venn diagram shows the overlapping among UBN-H3.3, UBN1-H3.3, and UBN2-H3.3. **g**, **h** UBN1 and UBN2 are required for H3.3 deposition at promoters and enhancers. Meta-analysis of dynamic changes of H3.3 deposition at promoters (**g**) and enhancers (**h**) after HIRA, UBN1, or UBN2 knockout and double depletion. Reads were normalized to 10 million in each data set. **i**, **j** UBN-H3.3 marks more active promoters. Boxplots show the H3K27ac level (I) or gene expression (**j**) of UBN-H3.3 marked promoters. **: *p* < 0.01. (**k**, **l**) UBN-H3.3 marks more active enhancers. Boxplots show H3K27ac level (K) of UBN-H3.3 marked enhancers and expression level of the regulated genes (**l**). **: *p* < 0.01

To answer how HIRA complex contributes to the genome-wide distribution of H3.3 in vivo, we generated a series of stable mES cell lines carrying 3xFlag-HA tag in the C-terminus of *H3f3b, Hira*, *Ubn1* or *Ubn2* allele by CRISPR/Cas9-mediated knock-in technique (Additional file 1: Figure S4A). Genotyping and Western blot analyses verified the expressions of H3.3-Flag-HA, UBN1-Flag-HA, UBN2-Flag-HA, and HIRA-Flag-HA fusion proteins in the corresponding mES cell lines (Additional file 1: FigureS4B-S4D). To analyze the genome-wide distribution of H3.3 and the subunits of HIRA complex at high resolution, we performed Flag- or HA-tag chromatin immunoprecipitation followed by massively parallel sequencing (ChIP-seq) in the corresponding knock-in mES cells. We detected 51,608 peaks for H3.3-HA, 7125 peaks for HIRA-Flag, 32,086 peaks for UBN1-Flag, and 46,610 peaks for UBN2-Flag in non-repetitive genomic regions using MACS [41]. Genome-wide analysis showed that HIRA, UBN1, and UBN2 are comparably enriched in genic regions, including promoter, intron, exon, and TTS, and the genome-wide distribution patterns of them did not show much difference (Additional file 1: Figure S4E). 41.7% of UBN1 peaks and 39.3% of UBN2 peaks overlap with H3.3 peaks (Additional file 1: Figure S4F). Heatmap shows that H3.3, HIRA, UBN1, and UBN2 are well co-localized at the H3.3 peaks (Fig. 4a). As 69.7% of UBN1 peaks overlap with UBN2 peaks (Additional file 1: Figure S4F), we wondered whether they physically interact with each other. Co-IP of endogenous proteins in mES or exogenous proteins in HEK293T cells both showed that UBN1 does not bind UBN2, even in the presence of HIRA (Fig. 4b and Additional file 1: Figure S4G), suggesting that the UBN1-HIRA and UBN2-HIRA complexes are present independently in mES cells.

To explore the function of each subunit of HIRA complex in H3.3 deposition, HIRA, UBN1, and UBN2 were individually knocked out in the *H3f3b* Flag-HA knock-in mES cell line (Fig. 4c). We found that HIRA knockout resulted in decreased protein level of UBN1 and UBN2; vice versa, UBN1 or UBN2 depletion also led to decrease of HIRA protein (Fig. 4c), which is consistent with previous reports that overall stability of HIRA complex is dependent on its integrity [19, 22, 38]. However, H3.3 protein level did not change obviously after knockout of HIRA, UBN1, or UBN2 (Fig. 4c). Then we performed ChIP-seq analysis for H3.3 deposition in these mES cells. Overall, H3.3 levels decreased significantly at genome-wide after HIRA knockout (Fig. 4d and Additional file 1: Figure S5B). The effect of knocking out UBN1 or UBN2 alone on H3.3 deposition was not as significant as HIRA knockout (Fig. 4d and Additional file 1: Figure S5B). However, in “double depletion” mES cells (knocked down UBN1 with siRNA in UBN2 knockout cell line, Additional file 1: Figure S5A), H3.3 levels decreased more obviously than that in HIRA knockout mES cells. These results suggested that UBN1 and UBN2 can deposit H3.3 redundantly to certain genome regions (Fig. 4d and Additional file 1: Figure S5B). Moreover, when UBN1 is knocked out, 24984 H3.3 peaks were still detected. Among these peaks, 15,933 (63.8%) peaks overlap with the H3.3 peaks in WT cells and 9051 peaks appear as new peaks. Interestingly, we found that 6095 (67.3%) of these new H3.3 peaks resulted from UBN1 knockout co-localize with UBN2 peaks (Additional file 1: Figure S5C). The same trend was observed in UBN2 knockout cells. After UBN2 knockout, 14,280 H3.3 peaks were detected, 2106 (14.7%) of which are new H3.3 peaks compared with H3.3 peaks in WT cells. Similarly, 1877 (84.5%) of these new H3.3 peaks overlap with UBN1 peaks (Additional file 1: Figure S5D). These results suggested that UBN1 and UBN2 may compete with each other for H3.3 deposition. However, after HIRA knockout or double depletion of UBN1 and UBN2, only 2656 and 273 H3.3 peaks remained, respectively. This extensive H3.3 lost at genome-wide upon knockout of HIRA and UBN1/UBN2 may lead to global change of background of ChIP-seq and biased peak detection. Thus, to define the H3.3 loci regulated by each individual subunit of HIRA complex, we mainly focused on the H3.3 peaks called in WT cells and analyze the fold change of H3.3 read density upon knockout of different subunits of HIRA complex. HOMER (Hypergeometric Optimization of Motif EnRichment) [42] was used to quantitatively measure the fold change of H3.3, and with the threshold of 1.5-fold change, we identified 31,066, 13,578, 17,285, and 45,939 H3.3 peaks as downregulated H3.3 after HIRA knockout, UBN1 knockout, UBN2 knockout, and “double depletion”, respectively. We termed these H3.3 peaks as “HIRA-H3.3” (HIRA-dependent H3.3), “UBN1-H3.3” (UBN1-dependent H3.3), “UBN2-H3.3” (UBN2-dependent H3.3), and “UBN-H3.3” (UBN1/2 -dependent H3.3) for further use. As shown in Fig. 4e, more than half of UBN1-H3.3 peaks (54.2%) and UBN2-H3.3 peaks (64.0%) are non-overlapping, suggesting that UBN1 and UBN2 deposit H3.3 at distinct genomic regions. The number of HIRA-H3.3 is far greater than either UBN1-H3.3 or UBN2-H3.3 (Fig. 4e). As our biochemical results suggested that HIRA subunit cannot interact with H3.3 directly, it is likely that deposition of HIRA-H3.3 is mediated by either UBN1 or UBN2. Indeed, when both UBN1 and UBN2 are depleted, the downregulated H3.3 (UBN-H3.3) contains most of HIRA-H3.3 (Additional file 1: Figure S5E). Therefore, our biochemical results and bioinformatics results demonstrated that the deposition of HIRA-H3.3 was indeed mediated by UBN1/2 subunits. In addition, as shown in Fig. 4f, another 23100 H3.3 peaks are downregulated when both UBN1 and UBN2 are depleted, suggesting that these H3.3 regions are redundantly regulated by both the UBN1-HIRA and UBN2-HIRA complexes. Taken together, our results revealed that the UBN1-HIRA and UBN2-HIRA complexes deposit H3.3 at distinct genomic regions in a cooperative way in mES cells.

To analyze how UBN1-HIRA and UBN2-HIRA cooperatively regulate the deposition of H3.3,, we divided UBN1/2-regulated H3.3 regions into four groups as indicated in the Venn diagram of Additional file 1: Figure S5F: I, UBN1-specific; II, Either; III, UBN2-specific; IV, Double-specific (Additional file 1: Figure S5F). Then we analyzed the chromatin states of these four H3.3 regions. We found that group IV H3.3 regions are more enriched with the active markers, including H3K4me3, H3K27ac, and DNaseI (Additional file 1: Figure S5G). On the contrary, group II H3.3 regions have a relative higher level of repressive markers, e.g. H3K9me3 and H3K27me3. Group I and group III H3.3 regions show intermediate level of active and repressive markers (Additional file 1: Figure S5G). These results suggest that UBN1 and UBN2 may redundantly deposit H3.3 at euchromatin regions and cooperatively deposit H3.3 at heterochromatin regions. Interestingly, we also found that active enhancers are more enriched in group IV H3.3 regions and poised enhancers are more enriched in group II H3.3 regions. However, the two promoter states (active and poised) did not show a preference to any H3.3 groups (Additional file 1: Figure S5H).

### UBN1 and UBN2 are required for H3.3 deposition at promoters and enhancers

Previously, we and others have reported that H3.3 is enriched at active gene bodies and regulatory regions, particularly at promoters and enhancers [12, 36]. However, it is still a puzzle how H3.3 deposition is regulated at these genomic regions. We therefore went on analyzing the dynamic changes of H3.3 at these regions upon depletions of HIRA, UBN1, UBN2, and “double depletion”. Although knockout of either UBN1 or UBN2 showed less obvious effects on H3.3 deposition compared with HIRA knockout, double depletion of UBN1 and UBN2 resulted in a considerable reduction of H3.3 at gene bodies and promoters (Additional file 1: Figure S5I, Fig. 4g). As for the enhancer regions, either UBN1 or UBN2 knockout only resulted in mild decrease of H3.3, which is similar to that observed after HIRA knockout (Fig. 4h). However, “double depletion” resulted in severe reduction of H3.3 (Fig. 4h), indicating that the UBN1-HIRA and UBN2-HIRA complexes are also responsible for H3.3 deposition at enhancers. To understand the biological function of H3.3 deposited at these *cis*-regulatory regions, we analyzed the correlation between H3.3 levels at these regions and transcriptional activities. According to epigenetic marks, promoters were classified into active promoters (marked by H3K4me3 but not by H3K27me3), bivalent promoters (marked by both H3K4me3 and H3K27me3), and other promoters (including repressive promoters marked by H3K27me3 but not H3K4me3 and those promoters without H3K4me3 or H3K27me3) [43, 44]. We found that UBN-H3.3-marked promoters are enriched in active promoters compare with total promoters (Additional file 1: Figure S5J). Further analysis demonstrated that UBN-H3.3-marked promoters display higher levels of H3K27ac and gene expression (Fig. 4i, j), suggesting that the HIRA complexes tend to deposit H3.3 at more active promoters. Similarly, enhancers are also classified into active enhancers (marked by both H3K4me1 and H3K27ac) and poised enhancers (marked by H3K4me1 but not H3K27ac) [45]. We found that UBN-H3.3-marked enhancers are more enriched in active enhancers (Additional file 1: Figure S5K). Further analysis showed that UBN-H3.3 marked enhancers exhibit higher levels of H3K27ac (Fig. 4k), suggesting UBN-H3.3 preferentially marks more active enhancers. To test whether enhancers marked by UBN-H3.3 display higher regulatory activity, the enhancers were assigned to the closest genes as reported [45]. As shown in Fig. 4l, genes associated with UBN-H3.3-marked enhancers show higher expression levels than those genes associated with enhancers without H3.3 peaks. However, after double depletion of UBN1 and UBN2 in mES cells, only 52 genes were downregulated and 29 genes were upregulated (Additional file 1: Figure S5L). Taken together, our results demonstrate that the UBN1-HIRA and UBN2-HIRA complexes are required for H3.3 deposition at *cis*-regulatory elements, including active promoters and active enhancers. However, even H3.3 evidently marks more active promoters and enhancers, it is not necessary for maintaining the transcriptionally active states of targeted genes in mES cells.

### Phe/Ile/Asp amino acid residues of UBN1 and UBN2 are involved in the binding and deposition of H3.3

**Fig. 5:**
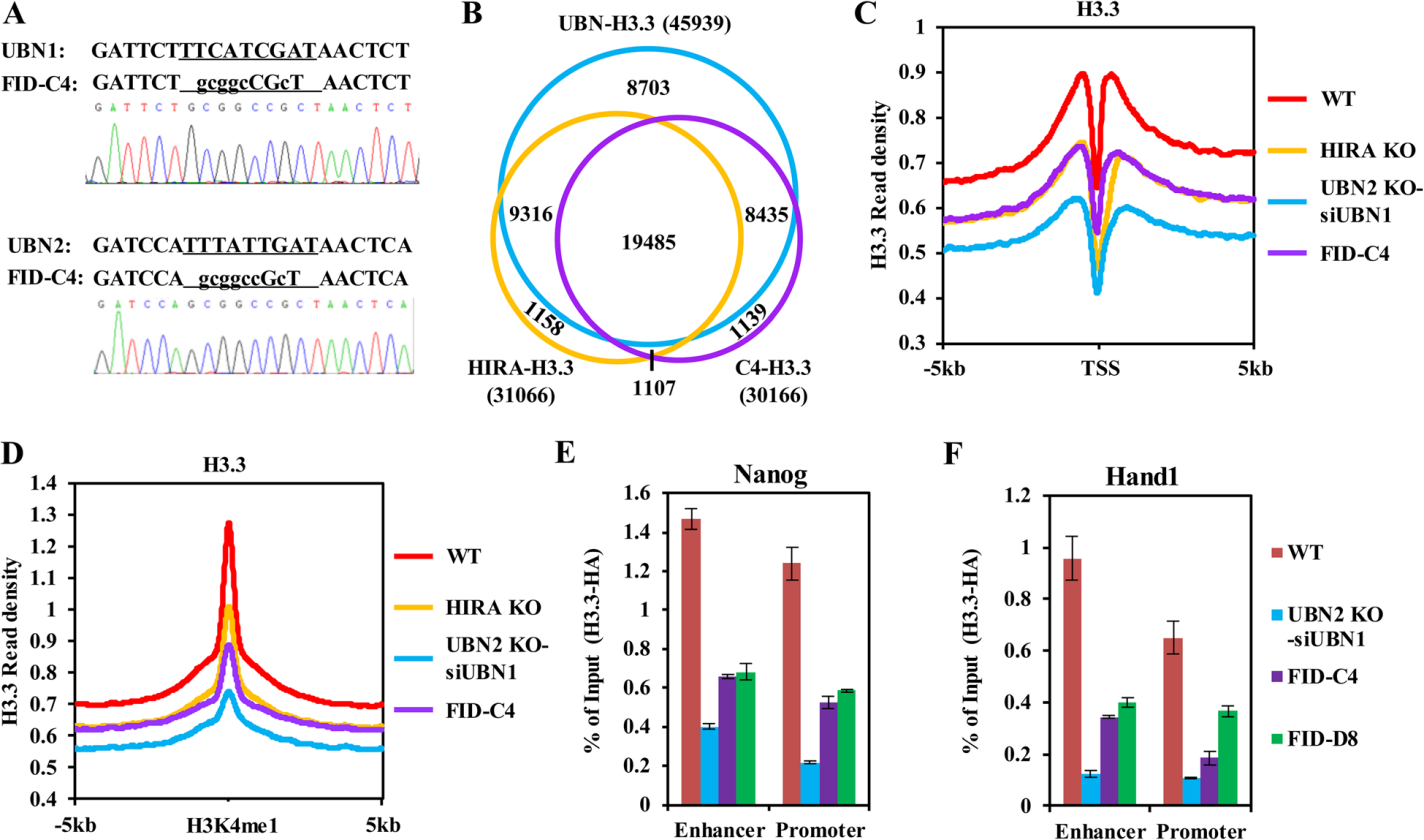
Phe/Ile/Asp amino acid residues of UBN1 and UBN2 are involved in the binding and deposition of H3.3. **a** Sanger sequencing of PCR product shows that Phe/Ile/Asp of both UBN1 and UBN2 were mutated to Alanine in FID-C4 cell line. **b** Venn diagram shows the overlapping among C4-H3.3, UBN-H3.3, and HIRA-H3.3. **c**, **d** Phe/Ile/Asp amino acids of UBN1/2 were important for the deposition of H3.3. Meta-analysis shows that alternation of the deposition of H3.3 at both promoters (**c**) and enhancers (**d**) in mES cells with FID/AAA mutation of UBN1 and UBN2. Reads were normalized to 10 million in each data set. (**e**, **f**) ChIP-qPCR analysis of H3.3 at enhancers and promoters of active (Nanog) (**e**) and bivalent (Hand1) (**f**) genes. The qPCR value was normalized to 1% input of each sample. Standard deviation was derived from three replicates

Recently, it has been reported that the HRD domain of UBN1 binds H3.3 independent of HIRA subunit, with several amino acid residues in this domain critical for this interaction in vitro, including Phe138, Ile139, and Asp140 [35]. Knockout of UBN1 or UBN2 may lead to loss of functions mediated by the interaction between UBN1 or UBN2 and other binding proteins; therefore, the chaperone activity-deficient mutation of UBN1 or UBN2 will provide a specific tool to explicitly study the deposition and function of H3.3 that mediated by HIRA complexes in vivo. To this end, we first verified the function of these residues in the recognition of H3.3 by UBN1 using co-IP assay. We showed that mutation of these three residues to Alanine abolished the interaction between Flag-UBN1 and HA-H3.3, but not the interaction between Flag-UBN1 and Myc-HIRA (Additional file 1: Figure S6A). To further investigate the biological function of these sites in H3.3 deposition in mES cells, we introduced both F138A/I139A/D140A mutations of UBN1 and F215A/I216A/D217A mutations of UBN2 by CRISPR/Cas9 in the *H3f3b* Flag-HA knock-in mES cells. Two cell lines carrying these mutations were used in this study, termed “FID-C4” and “FID-D8”, and these mutations were confirmed by Sanger sequencing (Fig. 5a). Then we immunoprecipitate H3.3 by HA tag, and found that H3.3-HA cannot interact with HIRA subunit in FID-C4 cells (Additional file 1: Figure S6B), confirming the conclusion that HIRA subunit does not bind H3.3 directly and the interaction between HIRA and H3.3 is dependent on UBN1/2. ChIP-seq analysis revealed that 30,166 H3.3 peaks were downregulated in the FID-C4 cells, 92.6% of which overlapped with UBN-H3.3 peaks (Fig. 5b). These results indicated that the Phe/Ile/Asp residues of UBN1 and UBN2 are indeed important for the recognition and deposition of H3.3 by HIRA complexes in vivo. As 39.2% UBN-H3.3 peaks remain unaffected by these mutations, other amino acid residues or regions of UBN1/2 may be involved in the recognition and deposition of H3.3. In addition, we further demonstrated that the FID/AAA mutations of UBN1 and UBN2 resulted in a significant reduction of H3.3 deposition at both promoters and enhancers (Fig. 5c, d). Genome tracks show the reduction of H3.3 at promoters and enhancers of an active gene Nanog and a bivalent gene Hand1 after UBN1/2 double depletion or FID/AAA mutation (Additional file 1: Figure S6C). ChIP-qPCR of H3.3 validates the role of HIRA complexes in H3.3 deposition at these genomic regions (Fig. 5e, f). Together, these results suggested that Phe/Ile/Asp amino acid residues of UBN1 and UBN2 are involved in the binding and deposition of H3.3.

### UBN1- and UBN2-mediated H3.3 deposition is involved in neural progenitor cell differentiation

**Fig. 6:**
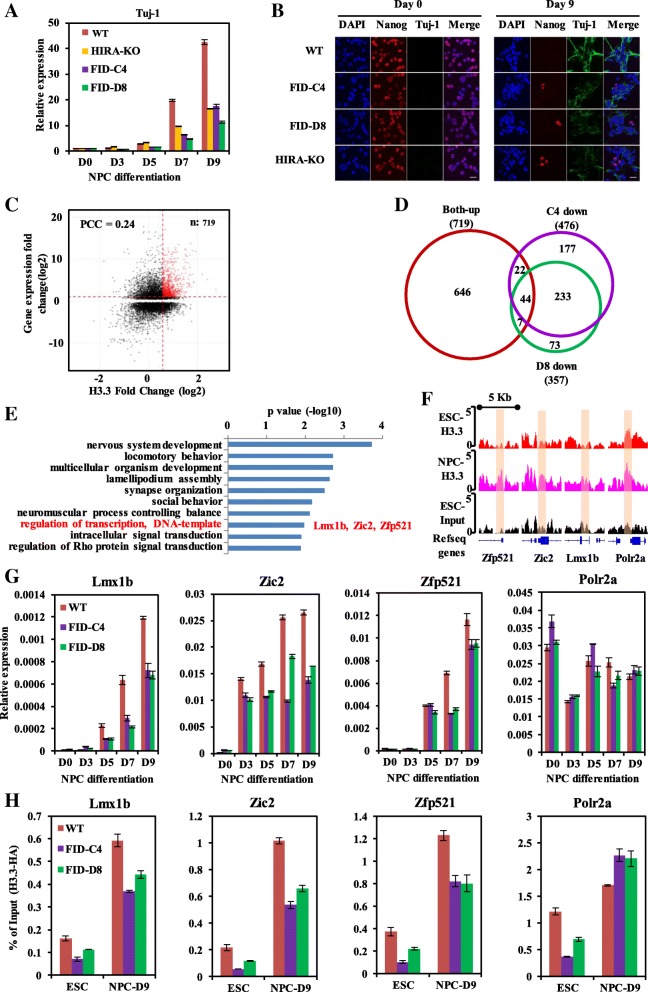
UBN1- and UBN2-mediated H3.3 deposition is involved in neuron progenitor cell differentiation. **a** RT-qPCR of Tuj-1 in WT and FID/AAA-mutated mES cells during NPC differentiation. The expression level of Tuj1 was normalized to GAPDH and day 0. **b** Immunofluorescence shows expression of Nanog and Tuj-1 in WT and FID/AAA-mutated cells during NPC differentiation. Scale bar, 20 μm. **c** Dot plot shows the relationship between the dynamics of H3.3 at promoter regions and gene expression during NPC differentiation. H3.3 read density was counted within promoters (TSS ± 500 bp) to calculate fold change from ES cell to NPC by HOMER. The Red dots represent the genes with upregulated expression and increased H3.3 signal at promoters. The thresholds for H3.3 fold change and gene expression are 1.5 and 2 respectively. PCC, Pearson’s correlation coefficient. **d** Venn diagram shows the relationship between the “both-up” genes and downregulated genes in NPCs derived from FID/AAA-mutated mES cells. **e** The top 10 enriched biological function terms from GO analysis of the 44 genes by DAVID. Lmx1b, Zic2, and Zfp521 are the enriched genes related to “regulation of transcription, DNA-templated”. A full list of significantly enriched terms is in Additional file 1: Figure S6E. **f** Genome tracks show H3.3 deposition at promoters of Zfp152, Zic2, Lmx1b, and Polr2a during NPC differentiation of WT mES cells. **g** RT-qPCR of gene expression of Zfp152, Zic2, Lmx1b, and Polr2a during NPC differentiation of WT and FID/AAA-mutated mES cells. The gene expression levels were normalized to GAPDH. Standard deviations were derived from three replicates. **h** ChIP-qPCR of H3.3 deposition at the promoters of Zfp152, Zic2, Lmx1b, and Polr2a during NPC differentiation in WT and FID/AAA-mutated cells. The qPCR value was normalized to 1% input of each sample. Standard deviation was derived from three replicates

HIRA complex and H3.3 have been shown to be involved in the regulation of development at multiple stages [23]. We therefore tested whether UBN1 and UBN2 regulated the deposition of H3.3 at *cis*-regulatory regions takes part in the cell differentiation process. We differentiated mES cell to neural progenitor cell (NPC) by N2B27 induction in vitro as previously reported [46]. As shown in Fig. 6a and Fig. 6b, FID/AAA mutations of UBN1 and UBN2 resulted in severe defects in NPC differentiation as indicated by the impairment of the expression of Tuj1 by RT-qPCR (Fig. 6a) and immunofluorescence (Fig. 6b). To further study the function of H3.3 during neural differentiation, we performed HA-H3.3 ChIP-seq and RNA-seq during NPC differentiation in WT mES cells. It turned out that positive correlations between the fold-change of gene expression and the fold-change of H3.3 deposition counted within promoter regions are observed in a subset of genes, including 719 genes whose gene expression and H3.3 deposition at promoter are both increased (termed as “both-up” genes) (Fig. 6c). RNA-seq analysis showed that compared with NPC cells from WT mES cells, 476 and 357 genes were found to be significantly downregulated in NPC cells from FID-C4 and FID-C8 mES cells respectively, and these genes significantly overlapped with each other (Fig. 6d). Of the 719 “both-up” genes, 44 were found to be downregulated in both NPCs from FID-C4 and FID-C8 mES cells (Fig. 6d). DAVID (Database for Annotation, Visualization and Integrated Discovery) [47] analysis showed that the enriched terms (*p* < 0.05) of these 44 genes are highly related to nervous system development (Fig. 6e). To obtain a more straightforward functional implication, the 2560 upregulated genes during NPC differentiation was used as background control during DAVID analysis (Additional file 1: Figure S6D). These results indicated that the UBN1-HIRA and UBN2-HIRA complexes mediate H3.3 depositions at regulatory regions of genes involved in neural differentiation. Interestingly, enriched genes include a few transcription factors that have been previously reported to be important for the development of the nervous system under the term of “regulation of transcription, DNA-templated”, such as Lmx1b [48], Zic2 [49, 50], and Zfp521 [51]. More importantly, H3.3 levels at the promoters of these neural genes increased obviously during NPC differentiation, while it remained unchanged at the promoters of housekeeping genes (such as Polr2a) (Fig. 6f). RT-qPCR analysis further confirmed that the activation of these neural genes is deficient during NPC differentiation of mES cells with FID/AAA mutations (Fig. 6g). To analyze whether the defect of activation is related to the reduction of H3.3 deposition, H3.3 deposition at the promoter regions of these neural genes was explored using ChIP-qPCR assay. As shown in Fig. 6h, H3.3 level at promoters of Lmx1b, Zic2, and Zfp521 increased steadily during the differentiation of WT mES cells, which is consistent with our ChIP-seq data. However, the deposition of H3.3 was impaired during NPC differentiation of mES cells with FID/AAA mutations (Fig. 6h). Together, these results suggested that the UBN1-HIRA and UBN2-HIRA complex-mediated H3.3 depositions at promoters of developmental genes are involved in gene activation during NPC differentiation. Furthermore, our results suggested that the FID/AAA mutants can be used as a precise tool to explore the function of H3.3 in vivo without disrupting the other possible function of UBN1/UBN2 proteins.

## Discussion

Previously, we showed that H3.3 is preferentially deposited at *cis*-regulatory elements, such as enhancers, to prime for the activation of RAR/RXR-targeted genes in mES cells [36]. However, it is still largely uncharacterized how histone variant H3.3 is specifically deposited at these enhancers and what roles it plays in regulation of gene transcription during cell differentiation.

### UBN1 and UBN2 subunits of HIRA complexes specifically recognize and directly bind to H3.3

The H3.3 variant differs from canonical H3.1 by only five amino acid residues, yet the HIRA complexes and the DAXX/ATRX complex can specifically recognize and bind to H3.3 rather than the canonical H3.1. Recently, we and others have shown that two residues of H3.3, Ala87, and Gly90, are the principal determinants for specific recognition and binding by DAXX [17, 18]. The HIRA complex is identified as the first chaperone that can specifically bind and deposit H3.3 [9]. In mammalian cells, HIRA complex is composed of three core subunits: HIRA, UBN1, and Cabin1 [9, 19]. None of these three subunits shares sequence homology with DAXX, thus the HIRA complex may utilize a different way to recognize and bind H3.3. Of note, a recent report showed that the HRD domain of UBN1 preferentially binds H3.3 independent of HIRA subunit, with several amino acid residues in this domain are critical for this interaction in vitro, including Phe138, Ile139, and Asp140 [35]. Here, we utilized biochemical methods, cell-based targeting assays and genomic approaches to illuminate that both UBN1 and UBN2 specifically recognize and directly bind H3.3. We further confirmed that Phe/Ile/Asp residues of UBN1 and UBN2 are indeed involved in the binding and deposition of H3.3 in vivo through biochemical assays and bioinformatics analysis, and revealed an explicit role of the HIRA complex-mediated H3.3 in neural differentiation.

HIRA subunit was originally identified as a histone-binding protein which was responsible for a nucleosome assembly pathway independent of DNA synthesis [37, 52, 53]. In addition, HIRA protein not only interacts with HRD domain of UBN1 through its WD repeat domain [19], but also binds the N terminus of Cabin1 through its C terminus [22]. Thus, HIRA protein is regarded as a scaffold protein for HIRA complex through bringing together UBN1 and Cabin1. Our results demonstrated that HIRA subunit is unable to bind H3.3 directly, while it enhances the binding affinity of UBN1 toward H3.3. In addition, HIRA subunit was reported to stabilize the whole HIRA complex, including UBN1 and Cabin1 [38, 54], and we also observed that the protein levels of both UBN1 and UBN2 are decreased after HIRA knockout (Fig. 4c). These results indicate that HIRA subunit regulates H3.3 deposition onto chromatin through stabilizing UBN1 or UBN2 subunit and enhancing its binding toward H3.3 as well, which may explain why HIRA subunit is also essential for H3.3 deposition in vivo during mammalian embryo development [23]. The WD repeat domain of HIRA was found to be sufficient for binding UBN1 [19]; however, we found that both the WD repeat domain and the B domain of HIRA subunit are required for the UBN1-dependent interaction between HIRA subunit and H3.3. As the B domain of HIRA binds histone chaperone Asf1a, which is able to bind histone variant H3.3 as well [55], it is possible that the interaction between HIRA and H3.3 is mediate by Asf1a. To rule out this possibility, we tested the interaction between HIRA and H3.3 using the HIRA R459A/R460A mutant, which impairs its binding to Asf1a [56]. Our results showed that the interaction between HIRA subunit and H3.3 is not affected by the R459A/R460A mutation of HIRA, suggesting that this interaction is independent of Asf1a. To further explore the molecular mechanism of the interaction between the HIRA complex and H3.3, it will be necessary to determine the structure of the HIRA/UBN1/H3.3/H4 complex at atomic resolution.

To understand how the HIRA complex distinguishes H3.3 from canonical H3, we analyzed the key residues in H3.3 that are critical for UBN1 recognition and binding. Previously, we revealed that DAXX recognizes H3.3 mainly through two key resides, Ala87 and Gly90 of H3.3, and either one of them is sufficient to warrant the specific interaction between H3.3 and DAXX [17, 18]. Recently, it was reported that Gly90 of H3.3 is critical for the specific binding with HRD domain of UBN1 in vitro [35]. Interestingly, we found that both Gly90 and Ala87 of H3.3 are indispensable for the interaction with UBN1 in vivo, and swap Ser87 and Met90 of H3.1 to Ala87 and Gly90 of H3.3 is sufficient to confer the specific interaction between UBN1 and H3.1 mutant. The critical role of Ala87 is in agreement with the structural insight that the H3.3 Ala87 beta carbon forms van der Waals interactions with the beta and gamma carbons from UBN1 Met128 [35]. Therefore, it is reasonable to speculate that H3.3 carrying out either an Ala87Ser mutation or a Gly90Met mutation can only be recognized and deposited to heterochromatin regions by the DAXX/ATRX complex, but not to active euchromatin regions by the HIRA complexes. These mutants of H3.3 can be used as potential tools to distinguish the biological function of H3.3 at the different genome regions. Moreover, as both Ala87 and Gly90 residues are evolutionally conserved in histone variant H3.X and H3.Y [57], these H3 histone variants may also be chaperoned by the HIRA complex and DAXX/ATRX complex [58].

### The UBN1-HIRA and UBN2-HIRA complexes cooperatively deposit H3.3 at different genomic regions of mES cells

UBN1 is orthologous to Hpc2p in yeast, with a paralog—UBN2 in mammals [19, 21]. Our biochemical results showed that both UBN1 and UBN2 are expressed in mES cells, and UBN2 is able to specifically recognize and bind H3.3 in a similar way as UBN1 does. Consistently, our genome-wide analysis showed that the genome distribution of both UBN1 and UBN2 overlaps with H3.3 at high frequency in mES cells. During ChIP-seq analysis, the peak number of UBN1 and UBN2 is much larger than that of HIRA subunit. However, as the interaction between HIRA and H3.3 is mediated by UBN1/2, HIRA would bind to chromatin in a more loose and dynamic manner than UBN1/2 does, which could result in inefficient enrichment of HIRA-Flag at those regions during ChIP procedure. Thus, further biochemical evidences are required to test whether UBN1 and UBN2 can bind to chromatin independent of HIRA complex. More interestingly, we found that UBN1 and UBN2 can form distinct HIRA complexes, which are able to cooperatively deposit H3.3 both to their own and to common target genomic regions. Specifically, UBN1 and UBN2 may redundantly deposit H3.3 at euchromatin regions and cooperatively deposit H3.3 at heterochromatin regions.

H3.3 is enriched at the genic regions such as promoters and active transcribed gene bodies, and is also abundant at *cis*-regulatory regions, such as enhancer [12, 23, 36]. In this scenario, the UBN1-HIRA and UBN2-HIRA complexes may be involved in regulating chromatin-related biological processes through cooperative deposition of H3.3 to these regions. Indeed, we found that UBN1-HIRA and UBN2-HIRA complexes are responsible for H3.3 deposition at promoters, which is in agreement with the previous observation [12, 23]. We observe that knockout of UBN1 result in dramatic decrease of H3.3 level at the downstream of TSS but no obvious change at upstream of TSS. This asymmetric alternation of H3.3 level around TSS is also observed in other conditions of depleting of HIRA complex, including HIRA KO, UBN2 KO, and double depletion, although subtler compared with UBN1 KO. Given that gene transcription is asymmetric and unique directional, the relationship between asymmetric regulation of H3.3 by HIRA complex and directional transcription would be interesting to investigate further in future. Previously, we found that H3.3 is also highly enriched at enhancers to prime for the activation of RA-regulated genes [36]; however, how the deposition of H3.3 to enhancers is regulated is yet to be characterized. In this study, we found that UBN1 and UBN2 double depletion resulted in dramatic reduction of H3.3 at enhancers, which indicates that UBN1 and UBN2 are critical for H3.3 deposition at the enhancer regions. Thus, our results demonstrate that UBN1 and UBN2 of the HIRA complex are critical for H3.3 deposition not only at promoters but also at enhancers. Together, our results provide detailed molecular insights into how the deposition of H3.3 across the genome is regulated by the UBN1-HIRA and UBN2-HIRA complexes in mES cells.

### UBN1- and UBN2-mediated H3.3 deposition at *cis-*regulatory elements is critical for cell fate transition

H3.3 was generally regarded as an active mark of gene transcription [59]. However, it is still not fully understood what function H3.3 at the *cis*-regulatory elements actually plays in regulation of gene transcription. Our genome-wide analysis showed that the UBN1-HIRA and UBN2-HIRA complexes preferentially deposit H3.3 at more active promoters and more active enhancers. However, RNA-seq results showed that double depletions of UBN1 and UBN2 resulted in only modest effect on global gene expression in mES cells, which is consistent with the mild change of global gene expression after knockout of HIRA subunit [12] or depletion of H3.3 in mES cells [60]. These RNA-seq data suggest that deposition of H3.3 mediated by HIRA complexes at *cis*-regulatory elements is not sufficient for maintaining global transcription of its targeted genes in mES cells. However, HIRA complexes and H3.3 have been shown to play essential roles during development at multiple stages [23], and we also observed that disruption of chaperone activity of HIRA complexes toward H3.3 by FID/AAA mutations of UBN1 and UBN2 resulted in defects of neural differentiation of mES cells, which is consistent with the recent report that H3.3 is involved in neural stem cell differentiation [61]. Further analysis demonstrated that HIRA complex-mediated H3.3 deposition plays important roles in regulating the temporal expression of a group of developmental genes (especially transcription factors) that are essential for cell fate transition during neural differentiation of mES cells. Thus, our work provides mechanistic insights for the function of the HIRA complex-mediated H3.3 deposition during development. Furthermore, our bioinformatics and biochemical results suggested that the FID/AAA mutations of UBN1 or UBN2 are sufficient to suppress the deposition of H3.3 mediated by HIRA complexes, which provides a specific strategy to explore the function of H3.3 in vivo without disturbing the other possible function of HIRA or UBN1/2 proteins.

As discussed above, the active gene bodies and *cis*-regulatory regions are decorated with H3.3 by the HIRA complexes, while DAXX/ATRX complex has been shown to preferentially deposit H3.3 at repressive heterochromatin regions. Thus, active recruitment of HIRA complexes and DAXX/ATRX complex by additional factors could serve as a robust mechanism to ensure proper H3.3 deposition to their own targeted chromatin regions. Consistent with this idea, it was reported that Erythroid Krüppel-like factor (EKLF), an erythroid cell-specific transcription factor, can recruit HIRA complex to promoter of the adult β-globin gene through interacting with HIRA subunit [62]. Previously, we showed that H3.3 is able to impair higher-ordered chromatin folding and counteract H2A.Z-mediated chromatin compaction, and that it actively marks enhancers to prime transcriptional potential of retinoid acid-regulated genes via creating an open chromatin signature [36]. In a similar scenario, EKLF can also promote the expression of the adult β-globin gene through directing H3.3 deposition by the HIRA complex and regulating the chromatin structure at its promoter region [62]. More recently, it has been shown that replication protein A (RPA) physically interacts with HIRA subunit and regulates deposition of newly synthesized H3.3 to promoters and enhancers for gene regulation [63]. HIRA protein has also been reported to interact with other transcription factors or chromatin-binding factors, such as transcription factor RUNX1 [64], the histone methyltransferase Wolf-Hirschhorn syndrome candidate 1 (WHSC1) (NSD2/MMSET) [65], the SWI/SNF family chromatin remodeler CHD1 [66], BRG1/INI1 [67], and the Polycomb complex PRC2 [60]. In addition, UBN1 was originally defined as a ubiquitously expressed nuclear protein that interacts with EB1, a member of the basic leucine-zipper family of transcription factors [68], suggesting that UBN1 may also play important role in regulating the genomic localization of the HIRA complex. Considering the divergence and variability between UBN1 and UBN2 outside the HRD domain [19], it is of great importance to investigate how the UBN1-HIRA and UBN2-HIRA complexes are recruited to deposit H3.3 at distinct regulatory regions for gene regulation by various chromatin factors during cell fate transition.

## Conclusions

In this study, we found that the UBN1 and UBN2 subunits of the HIRA complexes specifically recognize and bind H3.3 directly, while the HIRA subunit enhances the binding affinity of UBN1 toward H3.3. UBN1 and UBN2 cooperatively deposit H3.3 at both active enhancers and active promoters in mES cells. However, UBN1- and UBN2-mediated deposition of H3.3 at these regulatory regions is not sufficient for maintaining the transcriptional activity of targeted genes. Additionally, disruption of histone chaperone activities of UBN1 and UBN2 by FID/AAA mutations results in the defect of H3.3 deposition at promoters of developmental genes which are involved in neural differentiation, and correlates with the failure of the activation of these genes during the neural differentiation from mES cells.

## Methods

### Cell culture and plasmids

Mouse ES cells were cultured in medium with 80% DMEM, 15% FBS, 1% nonessential amino acids, 1% 2-Mercaptoethanol, 1% L-glutamine, 1% nucleosides, 1% Pen/Strep, and 1000 U/ml leukemia inhibitory factor (LIF) in standard incubator with 5% CO2 at 37 °C. HEK293T cells and A03_1 cells were grown in DMEM and F-12 Ham’s medium, respectively, supplemented with 10% FBS in standard incubator with 5% CO2 at 37 °C.

The assay for neural progenitor cell differentiation was performed as described previously [46]. Briefly, the mES cells were plated onto 0.1% gelatin-coated plates at a density of 0.5–1 × 10^4^/cm^2^ in N2B27 medium. N2B27 medium is a 1:1 mixture of DMEM/F12 (Gibco, 11320-033) supplemented with N2 (Gibco, 17502-048) and Neurobasal medium (Gibco, 21103-049) supplemented with B27 (Gibco, 17504-044), 25 μg/ml insulin, and 50 μg/ml BSA. The medium was refreshed every other day during 9 days of culture.

Human UBN1, UBN2 (109-1315), HIRA, Asf1a, and DAXX were cloned from HeLa cDNA library. The Cabin1 plasmid was kindly provided by Dr. Yongjun Dang (Fudan University Shanghai Medical College). Single- or multiple-point mutations were introduced by standard mutagenesis PCR procedures, and mutations were confirmed by DNA sequencing.

### Genome editing of mES cells

To generate knockout ES cells, pX260 was modified to contain the guide sequence insert site of pX330 [69]. SpCas9 target sites on the exons were designed by the CRISPR design tool [70]. Plasmids were then transfected into mES cells by Lipofectamine 2000 (Invitrogen) according to the manufacturer’s instructions. Afterwards, cells were seeded into 10-cm dish by serious dilution, and 12 h later puromycin (InvivoGen) was used to select clones for 10 days. Then alone clones were picked out and screened by PCR followed by Sanger sequencing, and successful knockout was confirmed by Western blot.

To generate 3xFlag-HA knock-in cell lines, the pX260 with SpCas9 target site around the stop codon of target gene was constructed as for knockout. The donor plasmid containing the homologous arms for recombination was constructed as described [71]. The homologous arm containing the PAM sequence of SpCas9 target site was mutated to disrupt the PAM sequence. The donor plasmid and the pX260 plasmid were co-transfected into mES cells using Lipofectamine 2000 (Invitrogen) according to the manufacturer’s instructions. Next, cells were seeded into 10-cm dish at low density; 12 h later puromycin (InvivoGen) was added to select clones for 10 days. Clones were then picked out and screened by PCR and agarose gel electrophoresis. Clones with epitope tag insertion were then validated by Western blot and confirmed by PCR use primers spanning one of the homologous arms. To generate UBN1 and UBN2 double depletion cells, UBN1 was knocked down by siRNA oligonucleotide, the sequence used is 5′-GAUGCUGGAGGAAGAGAAA-3′. DNA sequence used for genome editing was provided in Additional file 2: Table S3.

### LacO-LacI targeting system

For LacO-LacI targeting experiments, A03_1 cells were grown on glass coverslips overnight. pmCherry-LacI-chaperones plasmids and EGFP-H3.1/H3.3 plasmids were co-transfected into cells with Lipofectamine 2000 (Invitrogen) according to the manufacturer’s instructions. Forty-eight hours after transfection, cells were washed with ice-cold PBS, fixed with 4% paraformaldehyde for 15 min at room temperature, then washed with PBS and stained with 4′,6-diamidino-2-phenylindole (DAPI) for 15 min. Fluorescent images were collected using an Olympus FV1000 microscope. The percentage of co-localizing cells was obtained by scoring at least 100 cells in each experiment.

### Co-immunoprecipitation

HEK293T cells were transfected with Flag-tagged and HA-tagged plasmids by Lipofectamine 2000 (Invitrogen) according to the manufacturer’s instructions. Cells were harvested 48 h after transfection and then were lysed in RIPA buffer (50 mM Tris-HCl pH 7.6, 150 mM NaCl, 1 mM EDTA, 0.1% Nonidet P-40, 10% glycerol). Whole-cell extracts were clarified by centrifugation for 20 min at 12,000×*g* at 4 °C. The supernatant was then incubated with anti-Flag M2 agarose beads (Sigma, A2220) overnight at 4 °C with gentle rocking. For immunoprecipitating the endogenous UBN1 or UBN2, the supernatant was incubated with in-house UBN1 antibody or in-house UBN2 antibody, then antibody with bind proteins were captured with protein-A agarose beads (Pierce, 20334). The beads were washed three times with 1 ml RIPA buffer and then denatured in 2× SDS-PAGE loading buffer before analysis of the proteins by immunoblotting. Antibodies used for Western blot were as follows: Asf1a (1:1000, Millipore, ABE149), Tubulin (1:3000, Sigma, T8203), FLAG (1:3000, Sigma, F7425), HA (1:3000, Sigma, H3663), Myc (1:2000, CWBIO, CW0299M), HIRA (1:500, Millipore, 04-1488), UBN1 (1:2000, Abcam, ab84953), UBN2 (1:2000, in-house antibody), H3.3 (1:1000, Millipore, 09–838), GAPDH (1:3000, Cell signaling, 5174), H3 (1;3000, Cell signaling, 4499). The unique peptides of mouse UBN1 and mouse UBN2 used to generated antibodies were UBN1 (CSAKAGVSKDAIVTGPAP) and UBN2 (PLPQREVSRAEPPMNQC). The in-house UBN1 and UBN2 antibodies were used for immunoprecipitation.

### GST pull-down assays

Recombinant GST-UBN1 (1–213) and GST-UBN2 (109–291) were purified from *E. coli* by GST tag; GST-HIRA (1–481) and His-UBN1 (1–213) proteins were co-expressed in Sf9 insect cells using a baculovirus expression system, and the complex was purified by GST tag.

To prepare H3-H4 tetramers, equal molar ratio of H3.1/H3.3 and H4 were mixed in a dialysis bag and dialyzed into refolding buffer (2 M NaCl, 10 mM Tris-HCl pH 7.5, 1 mM EDTA, 5 mM 2-mercaptoethanol) at 4 °C. After dialysis, the tetramers were purified by sizing column (Hiload Superdex200, Pharmacia) and analyzed by 15% SDS-PAGE gel.

For GST pull-down assays, GST fusion proteins were immobilized on Glutathione Sepharose 4 Fast Flow (GE Healthcare) resin, then histone tetramers were mixed with the resins in binding buffer (20 mM HEPES-HCl, pH 7.9, 500 mM NaCl, 1 mM EDTA, 5 mM 2-mercaptoethanol, 0.1% Nonidet P-40) overnight at 4 °C. The resins were washed five times with 1 ml binding buffer. The resin with bound proteins were boiled in 2× SDS Loading Buffer and separated on a 15% SDS-PAGE gel before staining with Coomassie Blue.

### Immunofluorescence

For immunofluorescence analysis of neural progenitor cell markers, cells were grown on glass coverslips coated by gelatin in N2B27 medium. To prepare samples, cells were washed twice with PBS and then fixed with 4% paraformaldehyde for 15 min, washed three times with PBS, and permeabilized with 0.4% Triton X-100 in PBS for 15 min at room temperature. Cells were incubated with 3% BSA in PBS for 1 h at room temperature and then incubated with primary antibodies (1:200, Nanog, A300-397A, BETHYL; tubulin-β3, 1:200, 801201, Biolegend) overnight at 4 °C. After three washes with PBS containing 0.1% Triton X-100, the cells were incubated with fluorophore conjugated secondary antibodies for 1 h and stained with DAPI for 15 min. Fluorescent images were collected on an Olympus FV1000 microscope.

### ChIP-qPCR and ChIP-seq

For chromatin immunoprecipitation (ChIP) analysis of H3.3, H3.3-Flag-HA knock-in mES cells were crosslinked with 1% formaldehyde in DMEM for 10 min at room temperature. For ChIP analysis of UBN1, UBN2, and HIRA subunits of HIRA complex, knock-in mES cells were first crosslinked with 2 mM Di-(*N*-succinimidyl) glutarate (DSG) in DPBS for 45 min at room temperature, then crosslinked with 1% formaldehyde in DMEM for 10 min at room temperature. Cells were then lysed in Buffer I (50 mM HEPES, 140 mM NaCl, 1 mM EDTA, 10% glycerol, 0.5% Nonidet P-40, 0.25% Triton X-100, protease inhibitors) for 10 min at 4 °C, then incubated in Buffer II (10 mM Tris-HCl, 200 mM NaCl, 1 mM EDTA, 0.5 mM EGTA, protease inhibitors) for 10 min at room temperature. Then the nuclei were re-suspended in Buffer III (10 mM Tris-HCl, 1 mM EDTA, 0.5 mM EGTA, 0.5% *N*-lauroyl-sarcosine, protease inhibitors) and were sonicated using a Bioruptor UCD-200 (Diagenode), and the fragment size of chromatin should be about 1000–2000 bp before crosslink reversion by agarose electrophoresis. Following crosslink reversal, the DNA fragment was about 100–500 bp as checked by agarose electrophoresis. During ChIP procedure, HA agarose resin (Sigma, A2095) or Flag agarose resin (Sigma, A2220) was blocked with 0.1% BSA and then incubated with 20–100 μg of sonicated chromatin in RIPE-150 buffer (50 mM Tris-HCl, 150 mM NaCl, 1 mM EDTA, 0.5% Triton X-100, protease inhibitors) overnight at 4 °C. For HA-ChIP, HA beads were then washed by RIPE-150 buffer for five times. For Flag-ChIP, Flag beads were washed five times in RIPE-500 buffer (50 mM Tris-HCl, 500 mM NaCl, 1 mM EDTA, 0.5% Triton X-100, and protease inhibitors). Chromatin was eluted using Direct Elution buffer (100 mM NaHCO3, 1% SDS). ChIP DNA was extracted using a standard phenol-chloroform extraction procedure. ChIP-qPCR was performed with SYBR dye (Roche) on an Applied Biosystems StepOnePlus system. For ChIP sequencing, libraries were prepared according to NEBNext Ultra DNA Library Prep Kit for Illumina (E7370L) and were sequenced using a HiSeq2000 system at Berry Genomics. The primers used for ChIP-qPCR analysis are in Additional file 1: Table S1.

### RNA preparation, RT-qPCR and poly(A) RNA-seq

RNA was extracted using Trizol (Invitrogen) according to the manufacturer’s instruction. For RT-qPCR, mRNA was reverse-transcribed to cDNA using a Perfect Real-Time kit (TaKaRa, RR047A). qPCR was performed with SYBR dye (Roche) using an Applied Biosystems StepOne Plus system. For poly(A) RNA-seq, libraries were prepared according to the Illumina TruSeq protocol and sequenced using a HiSeq2000 system at Berry Genomics. The primers used for RT-qPCR are listed in Additional file 1: Table S2.

### Sequencing data analysis

ChIP-seq clean reads were mapped to mm9 by Bowtie2 [72] with default parameters, and were filtered using samtools [73] to retain unique reads. Enriched peaks were detected using MACS2 [41]. The overlapping between peaks was analyzed with the BEDTools software [74]. HOMER [42] was used to annotate the peaks and count the read density at promoter, enhancer, and peak regions, and to quantitatively analyze the dynamic change of H3.3 after HIRA complex was disrupted. The read density was normalized to 10 million reads. RNA-seq clean reads were mapped to mm9 by TopHat [75]. The differential expression between samples was performed with Cuffdiff [76]. DAVID [47] was used to analysis the enriched gene functions in a gene group. IGV [77] was used to view the data tracks. Heat maps were generated using Java Treeview [78]; other plots were generated by R (http://www.r-project.org) or Microsoft Excel.

## Additional files

Additional file 1: **Figure S1.** The HRD domain is conserved in UBN1 and UBN2. **Figure S2.** UBN1 mediates the interaction between HIRA subunit and histone variant H3.3. **Figure S3.** Residues Ala87 and Gly90 of H3.3 are important for recognition and binding of H3.3 by HIRA complex. **Figure S4.** UBN1 and UBN2 co-exist in mESC. **Figure S5.** UBN1 and UBN2 cooperatively deposit H3.3 at *cis-*regulatory elements in mESC. **Figure S6.** UBN1- and UBN2-mediated H3.3 deposition is involved in neuron progenitor cell differentiation. **Table S1.** Primers used for ChIP-qPCR. **Table S2.** Primers used for real-time RT-qPCR. (DOCX 1184 kb)

Additional file 2: **Table S3.** Primers used for genome editing. (XLSX 11 kb)
