# Supplementary material for: UBN1/2 of HIRA complex is responsible for recognition and deposition of H3.3 at cis-regulatory elements of genes in mouse ES cells
Source: BMC Biol. 2018 Oct 3;16:110. doi: 10.1186/s12915-018-0573-9 (PMC6171237; doi:10.1186/s12915-018-0573-9)
Supplement: Supplementary file 1 — Figure S1. The HRD domain is conserved in UBN1 and UBN2. Figure S2. UBN1 mediates the interaction between HIRA subunit and histone variant H3.3. Figure S3. Residues Ala87 and Gly90 of H3.3 are important for recognition and binding of H3.3 by HIRA complex. Figure S4. UBN1 and UBN2 co-exist in mESC. Figure S5. UBN1 and UBN2 cooperatively deposit H3.3 at cis-regulatory elements in mESC. Figure S6. UBN1- and UBN2-mediated H3.3 deposition is involved in neuron progenitor cell differentiation. Table S1. Primers used for ChIP-qPCR. Table S2. Primers used for real-time RT-qPCR. (DOCX 1184 kb) [file 12915_2018_573_MOESM1_ESM.docx]

**UBN1/2 of HIRA complex is responsible for recognition and deposition of H3.3 at *cis*-regulatory elements in mouse ES cells**

Chaoyang Xiong^1, ¶^, Zengqi Wen^1, 2, ¶^, Juan Yu^1^, Jun Chen^1, 2^, Chao-Pei Liu^1^, Xiaodong Zhang^3^, Ping Chen^1^, Rui-Ming Xu^1, 2^, Guohong Li^1, 2, *^

**Supplemental Figures and Legends**

**
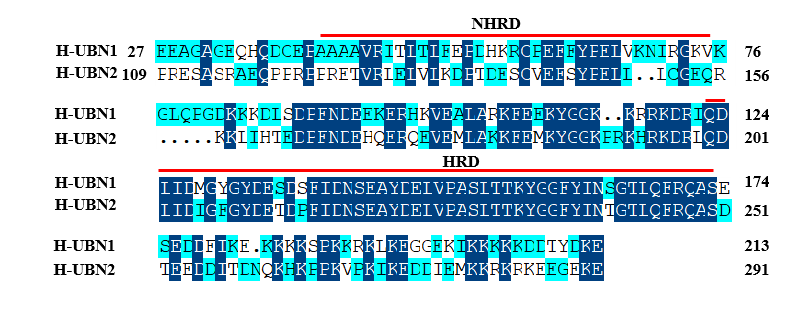
**

**Figure S1. The HRD domain is conserved in UBN1 and UBN2.**


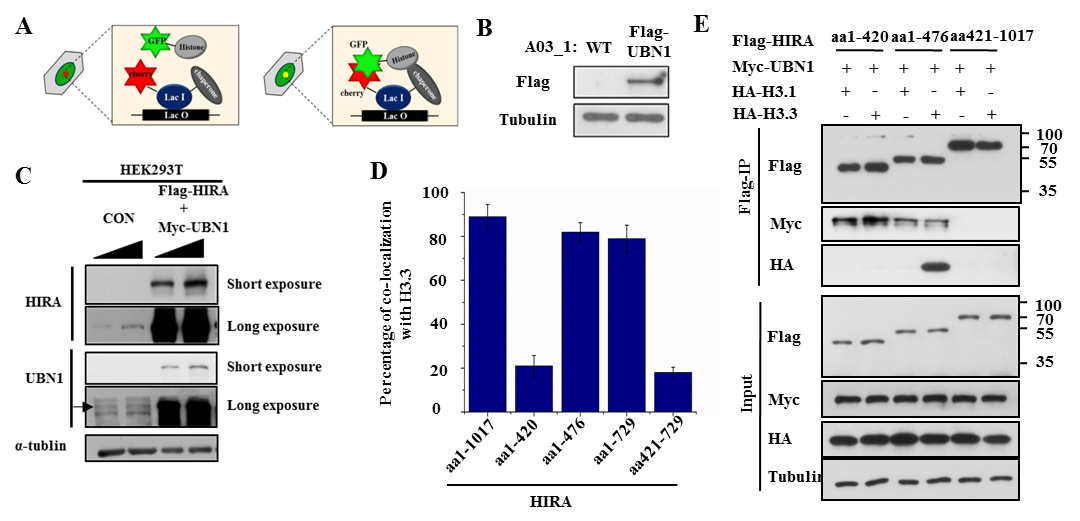


**Figure S2. UBN1 mediates the interaction between HIRA subunit and histone variant H3.3.** Related to Fig 2.

(A) A schematic diagram showing the LacO-LacI targeting system. The interaction between histones and chaperones results in the specific overlap of green and red focus.

(B) Western blot shows the expression of Flag-UBN1 in A03_1/Flag-UBN1 cell line.

(C) Western blot shows the protein levels of endogenous and exogenous HIRA and UBN1. Endogenous HIRA and UBN1 cannot be detected under short exposure time.

(D) Both WD repeat domain and B domain of HIRA are involved in the interaction with H3.3. Statistic results for Fig. 2C. About 100 cells in each experiment are counted.

(E) Interaction between truncation mutants of HIRA subunit and H3.3 is analyzed in the presence of UBN1 by Western blot analysis of anti-Flag immunoprecipitates.

^
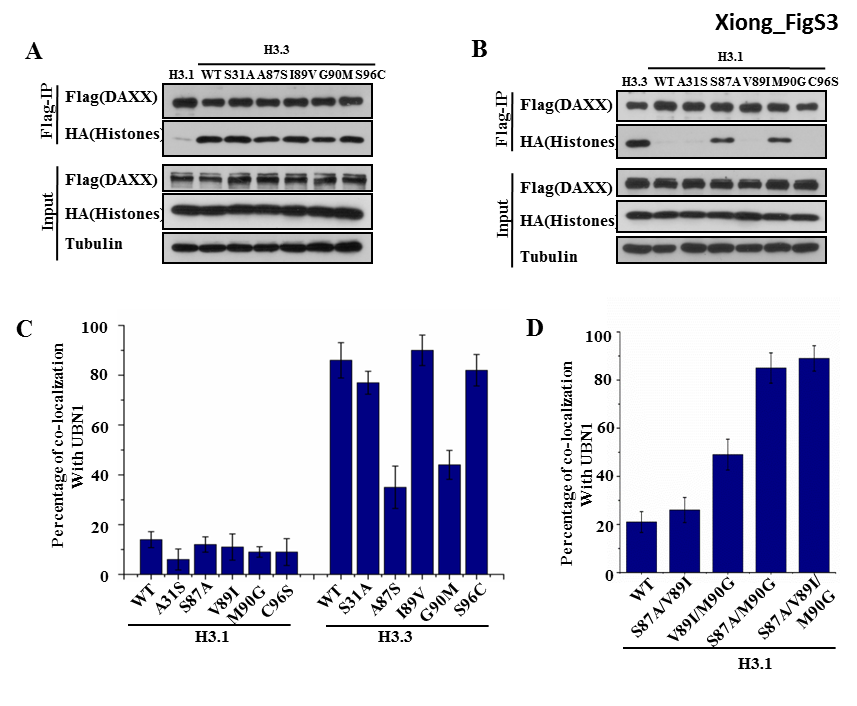
^

**Figure S3. Residues Ala87 and Gly90 of H3.3 are important for recognition and binding of H3.3 by HIRA complex.** Related to Figure 3.

(A) Single mutation of either Ala87 or Gly90 of H3.3 towards H3.1 is not sufficient to disrupt the interaction between DAXX and H3.3. Interaction between DAXX and H3.3 mutants is analyzed by Western blot analysis of anti-Flag immunoprecipitates.

(B) Single mutation of either Ser87 or Met90 of H3.1 towards H3.3 is sufficient to mediate the interaction between DAXX and H3.1. Interaction between DAXX and H3.1 mutants is analyzed by Western blot analysis of anti-Flag immunoprecipitates.

(C) Both Ala87 and Gly90 of H3.3 are required for bind UBN1. Statistics results for Fig.3A. About 100 cells in each experiment are counted.

(D) Ala87 and Gly90 of H3.3 are sufficient to confer the specificity toward UBN1. Statistics results for Fig.3C. About 100 cells in each experiment are counted.

^
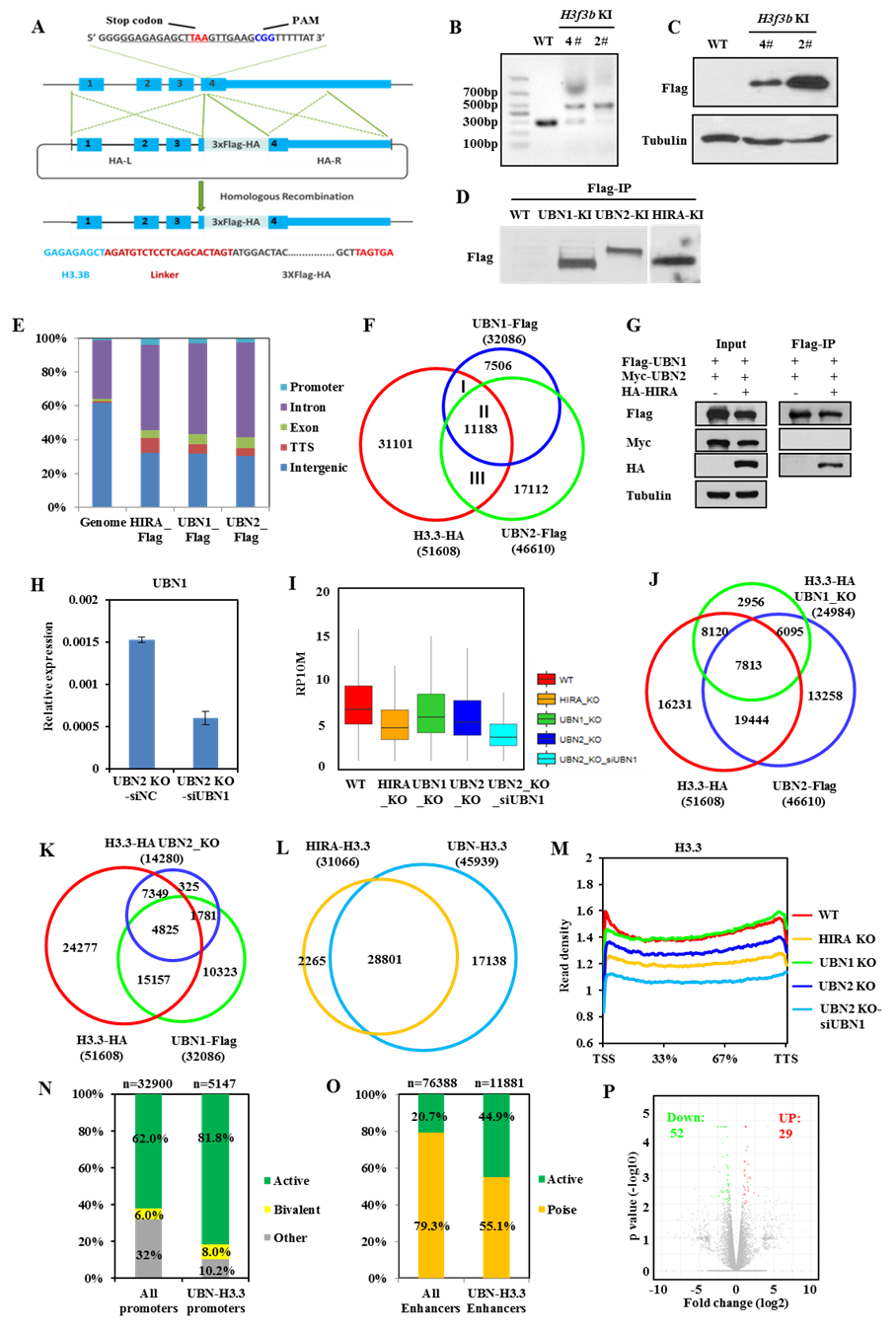
^

**Figure S4 UBN1 and UBN2 co-exist in mESC. Related to Fig 4.**

(A) Diagram shows H3.3 knockin strategy. HA-L, left homologous arm; HA-R: right homologous arm.

(B-C) PCR (B) and western blot (C) show H3.3 knockin clones. The H3f3b-KI-#2 clone with homozygote knockin alleles was used for downstream experiments.

(D) Flag IP shows knockin clones of UBN1, UBN2 and HIRA.

(E) Genome-wide distribution of HIRA-Flag, UBN1-Flag and UBN2-Flag.

(F) Venn diagram shows the overlapping among peaks of UBN1, UBN2 and H3.3.

(G) Interaction between UBN1 and UBN2 in the presence of HIRA is analyzed by Western blot analysis of anti-Flag .


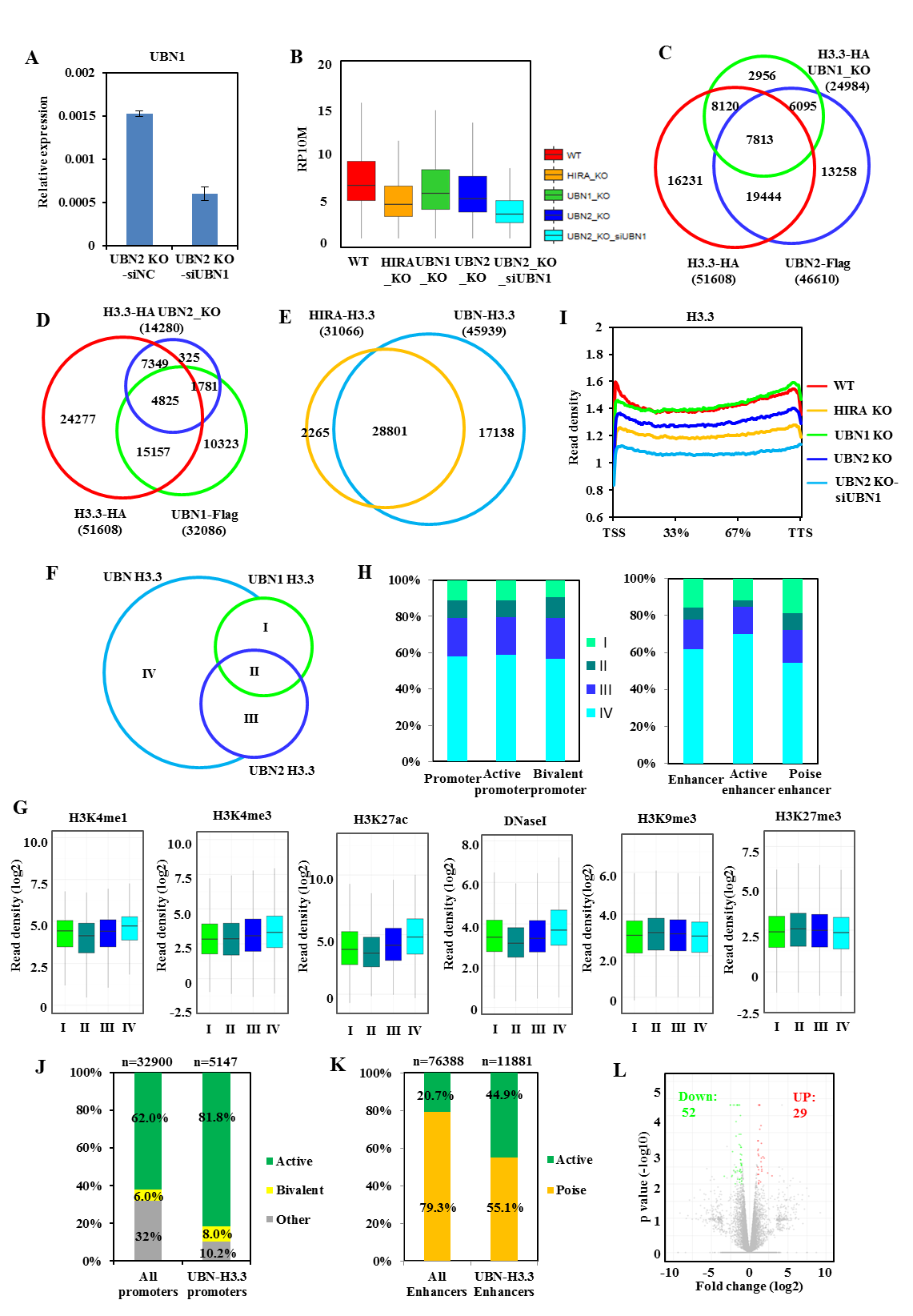


**Figure S5 UBN1 and UBN2 cooperatively deposit H3.3 at cis-regulatory elements in mESC. Related to Fig 4.**

(A) RT-qPCR shows UBN1 is knocked down in UBN2 knock out cells. The expression level was normalized to GAPDH.

(B) Box plot shows the decrease of H3.3 signal under each depletion condition of HIRA complex.

(C) Venn diagram shows the overlapping among WT H3.3, H3.3 detected after UBN1 KO and UBN2.

(D) Venn diagram shows the overlapping among WT H3.3, H3.3 detected after UBN2 KO and UBN1.

(E) Venn diagram shows the overlapping between HIRA-H3.3 and UBN-H3.3 peaks.

(F) Venn diagram shows the classification of H3.3 into four groups according to the regulation by UBN1/2.

(G) Boxplots show the epigenetic states of the four H3.3 groups.

(H) Bar plots show the distribution of the four H3.3 groups in promoter and enhancer.

(I) Meta-analysis shows that UBN1 and UBN2 are required for H3.3 deposition at gene bodies. Reads were normalized to 10 million in each data set.

(J) UBN-H3.3 marked promoters are enriched in active promoters.

(K) UBN-H3.3 marked enhancers are enriched in active enhancers.

(L) Volcano plot shows that 52 genes were down-regulated and 29 genes were up-regulated after double depletion. Differential expressed genes were identified by 2-fold change and p < 0.01.

**
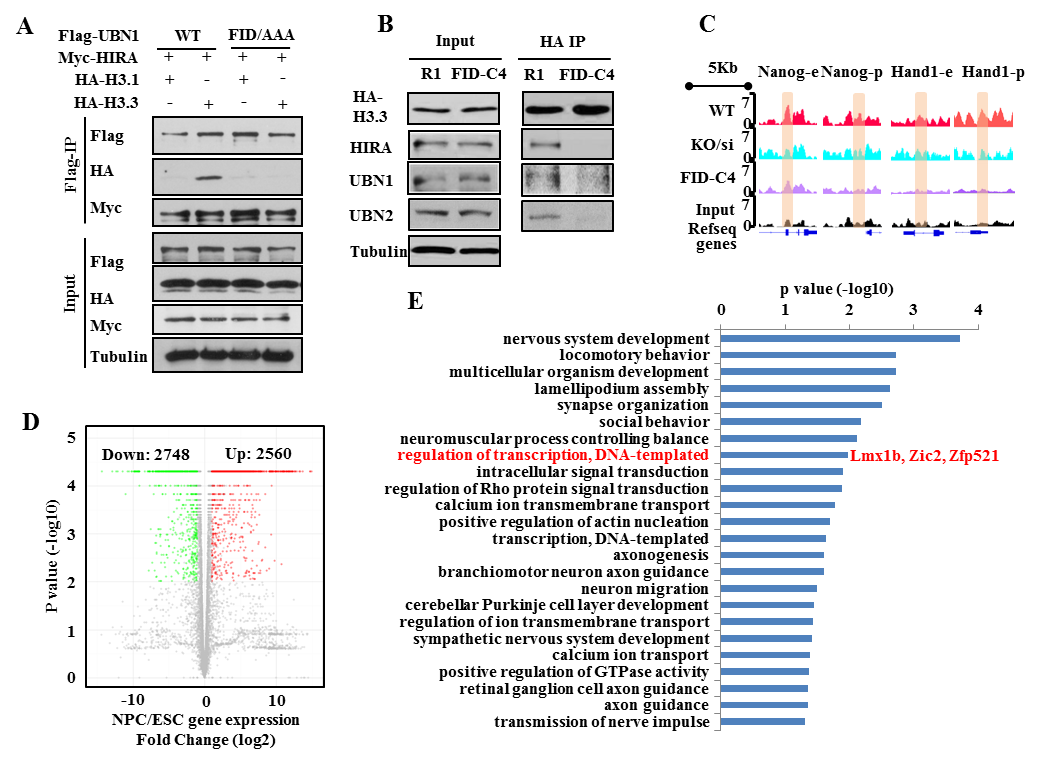
**

**Figure S6. UBN1 and UBN2 mediated H3.3 deposition is involved in neuron progenitor cell** differentiation. Related to Figure 5,6.

(A) UBN1-FID/AAA mutant cannot bind H3.3. Interaction between UBN1-FID/AAA mutant and H3.1, H3.3 in the presence of HIRA is analyzed by Western blot analysis of anti-Flag immunoprecipitates.

(B) HIRA subunit cannot interaction with H3.3 in FID-C4 cells.

(C) Genome tracks show H3.3 signal at enhancers and promoters of active (Nanog) and bivalent (Hand1) genes.

(D) Volcano plot shows that 2748 genes were down-regulated and 2560 genes were up-regulated during NPC differentiation of WT mES cells. Differential expressed genes were identified by 2-fold change and p < 0.01.

(E) Enriched biological function terms with p < 0.05 from GO analysis of the 44 genes by DAVID using the 2560 up-regulated genes during NPC differentiation of WT mES cell as control.

**Table S1. Primers used for ChIP-qPCR.**

| **Gene** | **Forward primer （5’-3’）** | **Reverse primer （5’-3’）** |
| --- | --- | --- |
| Hand1-E1 | ATGTGGTACCTCACCTTGGC | AGGGTGACTCTTACCCCTCA |
| Hand1-P1 | CTCTGGAAGTAAGGCCGCTC | GCAGCTACGCACATCATCAC |
| Lmx1b | CGATGCTGTTGGAACCTTCT | GGGTCTGGGTTCTAGTGCTG |
| Nanog-E1 | GCAGAAGTACCTCAGCCTCC | GACGTCCAGACGTTAGTTCG |
| Nanog-P1 | GCAGGACACAGGCTCTTTCT | CACGGCTTTCCTTTCAGCAC |
| Polr2a | CGACCGTGACAATCTGAGAA | GAATCACTGTGGTTGGCTGA |
| Zfp521 | GGAGGAGAAGGAGTGTGCTG | GCAGGCTGACAATGATTTCC |
| Zic2 | CACGAACTGTCTCCTGGTCA | AAGTCCCGGGTGGAATTAAA |

**Table S2. Primers used for real-time RT-qPCR.**

| **Gene** | **Forward primer （5’-3’）** | **Reverse primer （5’-3’）** |
| --- | --- | --- |
| Gapdh | GCTCACTGGCATGGCCTTCCG | GTGGGCCATGAGGTCCACCAC |
| Hand1 | CTACTTGATGGACGTGCTGG | CAACTCCCTTTTCCGCTTG |
| Lmx1b | TTCAAGGCATCCTTTGAGGT | GGCTTGACAGAACCTCTTGG |
| Nanog | GGCAGCCCTGATTCTTCTAC | CGCTTGCACTTCATCCTTT |
| Polr2a | CGCACCACGTCCAATGATAT | GTGCTGCTGCTTCCATAAGG |
| Tuj1 | CACCTATGGGGACCTCAACC | AGCCATCATGTTCTTGGCATC |
| UBN1 | TGGAGATTCTGGTGGTGGG | GAGTACATGAAGTGGAAATTGGGTT |
| UBN2 | ACAGCAGGAGCATCATTATTGGC | CCGTTCCAAAGCCAAGTAGC |
| Zfp521 | GCGAAACCGAGATCCCTCAA | TTGTGCTCTGTGATGTCGCT |
| Zic2 | CTGCTTCTGGGAGGAGTGTC | TCGAACTCACACTGGAAAGG |
